# Respiratory Subsets in Patients with Moderate to Severe Acute Respiratory Distress Syndrome for Early Prediction of Death †

**DOI:** 10.3390/jcm11195724

**Published:** 2022-09-27

**Authors:** Jesús Villar, Cristina Fernández, Jesús M. González-Martín, Carlos Ferrando, José M. Añón, Ana M. del Saz-Ortíz, Ana Díaz-Lamas, Ana Bueno-González, Lorena Fernández, Ana M. Domínguez-Berrot, Eduardo Peinado, David Andaluz-Ojeda, Elena González-Higueras, Anxela Vidal, M. Mar Fernández, Juan M. Mora-Ordoñez, Isabel Murcia, Concepción Tarancón, Eleuterio Merayo, Alba Pérez, Miguel A. Romera, Francisco Alba, David Pestaña, Pedro Rodríguez-Suárez, Rosa L. Fernández, Ewout W. Steyerberg, Lorenzo Berra, Arthur S. Slutsky, The Spanish Initiative for Epidemiology, Stratification and Therapies of ARDS (SIESTA) Network

**Affiliations:** 1 CIBER de Enfermedades Respiratorias, Instituto de Salud Carlos III, 28029 Madrid, Spain; 2 Research Unit, Hospital Universitario Dr. Negrín, 35019 Las Palmas de Gran Canaria, Spain; 3 Li Ka Shing Knowledge Institute, St. Michael’s Hospital, Toronto, ON M5B 1W8, Canada; 4 Surgical Intensive Care Unit, Dept. of Anesthesia, Hospital Clinic, IDIBAPS, 08036 Barcelona, Spain; 5 Intensive Care Unit, Hospital Universitario La Paz, IdiPaz, 28046 Madrid, Spain; 6 Intensive Care Unit, Hospital Universitario Virgen de Arrixaca, 30120 Murcia, Spain; 7 Intensive Care Unit, Hospital Universitario de A Coruña, 15006 La Coruña, Spain; 8 Intensive Care Unit, Hospital General Universitario de Ciudad Real, 13005 Ciudad Real, Spain; 9 Intensive Care Unit, Hospital Universitario Río Hortega, 47012 Valladolid, Spain; 10 Intensive Care Unit, Complejo Asistencial Universitario de León, 24001 León, Spain; 11 Intensive Care Unit, Hospital Universitario NS de Candelaria, 38010 Santa Cruz de Tenerife, Spain; 12 Intensive Care Unit, Hospital Clínico Universitario, 47003 Valladolid, Spain; 13 Intensive Care Unit, Hospital Universitario Virgen de La Luz, 16002 Cuenca, Spain; 14 Intensive Care Unit, Hospital Universitario Fundación Jiménez Díaz, 28040 Madrid, Spain; 15 Intensive Care Unit, Hospital Universitario Mutua Terrassa, Terrassa, 08221 Barcelona, Spain; 16 Intensive Care Unit, Hospital Universitario Regional Carlos Haya, 29010 Málaga, Spain; 17 Intensive Care Unit, Complejo Hospitalario Universitario de Albacete, 02006 Albacete, Spain; 18 Intensive Care Unit, Complejo Público Hospitalario de Zamora, 49022 Zamora, Spain; 19 Intensive Care Unit, Hospital El Bierzo, Ponferrada, 24404 León, Spain; 20 Department of Anesthesia, Hospital Universitario Río Hortega, 47012 Valladolid, Spain; 21 Intensive Care Unit, Hospital Universitario Puerta de Hierro, Majadahonda, 28222 Madrid, Spain; 22 Intensive Care Unit, Hospital General N.S. del Prado, 45600 Talavera de la Reina, Spain; 23 Department of Anesthesia, Hospital Universitario Ramón y Cajal, 28034 Madrid, Spain; 24 Department of Thoracic Surgery, Hospital Universitario Dr. Negrín, 35019 Las Palmas de Gran Canaria, Spain; 25 Department of Biomedical Data Sciences, Leiden University Medical Center, 2333 ZA Leiden, The Netherlands; 26 Department of Anesthesia, Critical Care & Pain Medicine, Massachusetts General Hospital, Boston, MA 02114, USA; 27 Interdepartmental Division of Critical Care Medicine, University of Toronto, Toronto, ON M5T3A1, Canada

**Keywords:** lung-protective ventilation, mortality, stratification, ARDS criteria, prediction, outcome

## Abstract

Introduction: In patients with acute respiratory distress syndrome (ARDS), the PaO_2_/FiO_2_ ratio at the time of ARDS diagnosis is weakly associated with mortality. We hypothesized that setting a PaO_2_/FiO_2_ threshold in 150 mm Hg at 24 h from moderate/severe ARDS diagnosis would improve predictions of death in the intensive care unit (ICU). Methods: We conducted an ancillary study in 1303 patients with moderate to severe ARDS managed with lung-protective ventilation enrolled consecutively in four prospective multicenter cohorts in a network of ICUs. The first three cohorts were pooled (*n* = 1000) as a testing cohort; the fourth cohort (*n* = 303) served as a confirmatory cohort. Based on the thresholds for PaO_2_/FiO_2_ (150 mm Hg) and positive end-expiratory pressure (PEEP) (10 cm H_2_O), the patients were classified into four possible subsets at baseline and at 24 h using a standardized PEEP-FiO_2_ approach: (I) PaO_2_/FiO_2_ ≥ 150 at PEEP < 10, (II) PaO_2_/FiO_2_ ≥ 150 at PEEP ≥ 10, (III) PaO_2_/FiO_2_ < 150 at PEEP < 10, and (IV) PaO_2_/FiO_2_ < 150 at PEEP ≥ 10. Primary outcome was death in the ICU. Results: ICU mortalities were similar in the testing and confirmatory cohorts (375/1000, 37.5% vs. 112/303, 37.0%, respectively). At baseline, most patients from the testing cohort (*n* = 792/1000, 79.2%) had a PaO_2_/FiO_2_ < 150, with similar mortality among the four subsets (*p* = 0.23). When assessed at 24 h, ICU mortality increased with an advance in the subset: 17.9%, 22.8%, 40.0%, and 49.3% (*p* < 0.0001). The findings were replicated in the confirmatory cohort (*p* < 0.0001). However, independent of the PEEP levels, patients with PaO_2_/FiO_2_ < 150 at 24 h followed a distinct 30-day ICU survival compared with patients with PaO_2_/FiO_2_ ≥ 150 (hazard ratio 2.8, 95% CI 2.2–3.5, *p* < 0.0001). Conclusions: Subsets based on PaO_2_/FiO_2_ thresholds of 150 mm Hg assessed after 24 h of moderate/severe ARDS diagnosis are clinically relevant for establishing prognosis, and are helpful for selecting adjunctive therapies for hypoxemia and for enrolling patients into therapeutic trials.

## 1. Introduction

Acute respiratory distress syndrome (ARDS) is a clinical-pathological entity [1] that is currently diagnosed using clinical and radiologic criteria [2,3] with poor accuracy and the low inter-rater reliability of clinicians [4,5]. Patients sharing the ARDS label differ in relation to the degree of lung injury and in their response to mechanical ventilation (MV) and adjunctive therapies [6]. The current definition of ARDS [2] accounts for the ratio of the partial pressure of arterial oxygen (PaO_2_) to the fraction of inspired oxygen (FiO_2_), or PaO_2_/FiO_2_, as a measure of hypoxemia at a positive end-expiratory pressure (PEEP) ≥ 5 cm H_2_O, regardless of the FiO_2_. In addition, the empirical cutoffs of baseline PaO_2_/FiO_2_ based on severity at 100, 200, and 300 mm Hg are somewhat arbitrary and have been inadequately validated [7,8,9,10,11]. 

The assessment of severity and prognosis in ARDS remains a challenge. The degree of hypoxemia in ARDS is a major determinant of the outcome [7,12], but the relationship between oxygenation and prognosis varies among published reports [7,8,9,10,13]. In ARDS, PEEP is applied to increase the lungs’ volume, keep the alveoli open, and improve oxygenation [14]. When PEEP recruits collapsed alveoli, the compliance of the respiratory system improves and increases the PaO_2_, although hypoxemia may coexist with minimally impaired lung compliance [15]. There is wide variation in the practice of choosing PEEP, but most patients with moderate to severe ARDS are managed with PEEP at ≥10 cm H_2_O in the first days of MV [9,16]. The progression of lung severity and the prognosis of ARDS has been reported to be related to changes in the PaO_2_/FiO_2_ in response to PEEP ≥ 10 [9,12]. Early identification of ARDS patients at a high risk of death after diagnosis would allow prompt escalation of therapeutic interventions, individualization of care, and precision in designing randomized clinical trials (RCTs). The challenges of the current definition of ARDS, which includes patients with different degrees of severity, make it difficult to successfully perform RCTs with positive findings that are highly generalizable [17]. 

In patients with ARDS, the PaO_2_/FiO_2_ ratio at ARDS onset/diagnosis is weakly associated with mortality. We hypothesized that setting a PaO_2_/FiO_2_ threshold of 150 mm Hg at 24 h from diagnosis of moderate/severe ARDS would improve predictions of the outcome in the intensive care unit (ICU). A PaO_2_/FiO_2_ threshold of 150 mm Hg has been used to identify patients for various interventions [18,19,20,21,22,23,24]. We previously reported a clinical classification system in a small population of 300 patients with moderate to severe ARDS to investigate whether the cutoff values of PaO_2_/FiO_2_ and PEEP would predict hospital mortality [25]. Boss et al. [26] validated the stratification model for hospital mortality in 519 ARDS patients, although the patients were not assessed with standardized ventilator settings. In this ancillary study, we have used a large population of moderate to severe ARDS patients to test and confirm whether the intersection of PaO_2_/FiO_2_ thresholds of 150 mm Hg and PEEP at 10 cm H_2_O could be useful for predicting ICU mortality and potential clinical translation.

## 2. Methods

This was an ancillary study using unrestricted data from our previously conducted and published studies [9,12,27,28] approved by the Ethics Committees of Hospital Universitario Dr. Negrín (Las Palmas de Gran Canaria, Spain), Hospital Virgen de la Luz (Cuenca, Spain), Hospital Clínico Universitario (Valladolid, Spain), and Hospital Universitario La Paz (Madrid, Spain), which have been adopted by all the participating centers, as required by Spanish legislation (see Supplementary File). The study was considered an audit, with waived informed consent (see the Supplementary File). The study followed the “Strengthening the Reporting of Observational Studies in Epidemiology” (STROBE) guidelines [29].

### 2.1. Patient Population, Study Design, and Oversight

We performed an ancillary analysis of the data derived from 1303 patients with moderate to severe ARDS treated with lung-protective MV included in our previously conducted and published studies [9,12,27,28]. The study was conducted in two steps (see the Supplementary File). First, we tested the classification model based on PaO_2_/FiO_2_ and PEEP cutoffs in 1000 patients admitted to a network of ICUs within the Spanish Initiative for Epidemiology, Stratification, and Therapies of ARDS (SIESTA) Program (see the Supplementary File). Data were pooled from three prospective observational multicenter cohorts (*n* = 300 patients in the ALIEN cohort, *n* = 300 patients in the STANDARDS cohort, and *n* = 400 patients in the STANDARDS-2 cohort) [9,12,27], enrolling consecutive patients managed with lung-protective MV (see the details in the Supplementary File) and who met the current criteria for moderate to severe ARDS [2], which included: (i) having an initiating clinical condition; (ii) symptoms developing within one week of a known clinical insult, or new or worsening respiratory symptoms; (iii) bilateral pulmonary infiltrates revealed by chest imaging (a chest radiograph or a computed tomography scan); (iv) the absence of left atrial hypertension or no clinical signs of left heart failure; and (v) hypoxemia, as defined by PaO_2_/FiO_2_ ≤ 200 mm Hg at PEEP ≥ 5 cm H_2_O regardless of the FiO_2_. Second, we confirmed the validity of the classification model in an independent cohort of 303 patients with moderate to severe ARDS managed with lung-protective MV who were included in a recent prospective observational multicenter study [28]. Each dataset had an adequate number of events (ICU deaths), as recommended [30].

All patients had arterial blood gases at study inclusion. We did not use SpO_2_ as a surrogate for PaO_2_ for enrolling patients. For identification of moderate/severe ARDS patients, the clinicians only considered qualifying blood gases while patients were clinically stable and did not consider transient falls in PaO_2_ resulting from acute events unrelated to the disease process (such as obstruction of the endotracheal tube by secretions, endotracheal suctioning, ventilator disconnection, or sudden pneumothorax, among others). No ICU patients meeting these criteria were excluded. We excluded patients younger than 18 years old; those with severe chronic pulmonary disease, acute cardiac failure, or brain death; patients with “do not resuscitate” orders; and postoperative patients receiving MV for <24 h (see the Supplementary File).

### 2.2. Data Collection and Outcomes

Day 0 was defined as the day and time when the patient first met the criteria for moderate to severe ARDS (see the Supplementary File). We collected information on the demographics, ARDS etiology, the Acute Physiology and Chronic Health Evaluation II (APACHE II) score [31], arterial blood gases, MV data, laboratory results, organ dysfunction (sequential organ failure assessment (SOFA) score) [32] at the onset of ARDS and after 24 h of treatment, and reported the primary cause of death in the ICU. We also recorded the duration of MV and calculated the number of ventilator-free days (VFDs) from the day of diagnosis of moderate/severe ARDS until Day 28 (see the Supplementary File). 

The attending clinicians followed the current guidelines for general critical care management, which included the following: (i) in case of sepsis, physicians were urged to ensure early identification of the causative microorganism, to start intravenous administration of antibiotics as soon as sepsis was suspected or recognized, and to optimize antibiotic selection and timely administration on the basis of antibiograms; (ii) fluid resuscitation and vasopressor use were individualized, with the goal of maintaining a systolic blood pressure ≥ 90 mm Hg or a mean arterial pressure ≥ 65 mm Hg; (iii) to maintain the hemoglobin between 7 to 10 g/dL (see the Supplementary File). For ventilatory management, the clinicians followed the current recommendations for lung-protective MV [33,34], with a tidal volume (VT) of 4–8 mL per kg of predicted body weight (PBW), a ventilatory rate to maintain PaCO_2_ at 35 to 50 mm Hg (permissive hypercapnia was allowed to target VT), a plateau pressure of <30 cm H_2_O, and PEEP and FiO_2_ combinations to maintain PaO_2_ > 60 mm Hg or SpO_2_ > 90% (see the details in the Supplementary File).

At the time of ARDS diagnosis, all patients were ventilated with PEEP ≥ 5 cm H_2_O, as mandated by the current ARDS definition [2]. For the purpose of this study, PaO_2_/FiO_2_ and Pplat at 24 h of enrollment were assessed using a standardized ventilatory setting with PEEP = 10 cm H_2_O and FiO_2_ = 0.5 [9,12]. When patients required PEEP > 10 or FiO_2_ > 0.5, a set of rules for setting PEEP and FiO_2_ were applied only during the standardized assessment, as described and validated previously by our group [9,12]. We did not exclude any patient ventilated with PEEP < 10 cm H_2_O at 24 h due to the absence of the site investigator or because the clinician determined that it was not in the best interest of the patient to apply these settings (see the Supplementary File). At baseline and at 24 h, patients were classified into four possible groups or subsets, based on the intersection of the PaO_2_/FiO_2_ and PEEP values: Subset I, patients with PaO_2_/FiO_2_ ≥ 150 mm Hg at PEEP < 10 cm H_2_O; Subset II, PaO_2_/FiO_2_ ≥ 150 at PEEP ≥ 10; Subset III, PaO_2_/FiO_2_ < 150 at PEEP < 10; and Subset IV, PaO_2_/FiO_2_ < 150 at PEEP ≥ 10. 

Patients were followed until ICU and hospital discharge. The primary outcome was all-cause ICU mortality. Secondary outcomes included the duration of MV, the number of VFDs up to Day 28 after moderate to severe ARDS diagnosis, and 30-day cumulative survival (see the Supplementary File).

### 2.3. Statistical Analysis

The plan of statistical analysis is provided in the Supplementary File. For the purpose of this study, we specified rules and expectations in advance [35] before the final statistical analyses were conducted (see the details in the Supplementary File). Quantitative variables are expressed as the means ± standard deviation (SD), and the median and 25th–75th percentiles (P_25_–P_75_). We used the Kolmogorov–Smirnov test to check for normal distribution of the data. We used Student’s *t* test or the Mann–Whitney test to compare two numerical variables, and ANOVA test to compare more than two numerical variables. We used Fisher’s exact test or Pearson’s Chi-squared test to check the relationships between categorical variables. We analyzed the probability of ICU survival to Day 30 for the initial four subsets and for the global subsets of patients with PaO_2_/FiO_2_ < 150 and ≥150 mm Hg at 24 h after moderate/severe ARDS diagnosis using the Kaplan–Meier method with the log-rank test. Patients discharged alive from the ICU before Day 30 of study inclusion were censored. No assumptions were made for missing data. We calculated the differences between the means, the risk ratio (RR), the odds ratio (OR), the hazard ratio (HR), and the 95% confidence intervals (CI). 

We used dot-plots to present the distributions of PaO_2_/FiO_2_ versus PEEP at ARDS diagnosis and at 24 h. We used a multivariable logistic regression analysis for adjusting the performance of PaO_2_/FiO_2_ < 150 mm Hg for predicting severity and ICU mortality in relation to each patient’s age and SOFA score. Patients’ age and SOFA scores are strong, well-known predictors of outcome in critically ill patients [36]. We performed a sensitivity analysis testing two combinations of assumptions by using two thresholds of PaO_2_/FiO_2_ (100 and 120 mm Hg). For all comparisons, a two-sided *p*-value < 0.005 was considered to keep the false discovery rate below 5%, as recently recommended [37]. Analyses were performed using R Core Team 2022 software, version 4.2 (R Foundation for Statistical Computing, Vienna, Austria).

## 3. Results

All-cause ICU mortality rates were similar in the testing and confirmatory cohorts (375/1000, 37.5% vs. 112/303, 37.0%, respectively) (*p* = 0.920). Pneumonia, sepsis, aspiration, and trauma were the most common risk factors associated with the development of moderate/severe ARDS (Table 1). At baseline, most patients from the testing and confirmatory cohorts met the Berlin criteria for moderate ARDS (590/1000, 59% vs. 196/303, 64.7%, respectively), and their overall ICU mortality rates were similar (203/590, 34.4% vs. 64/196, 32.6%, respectively; RR 1.05, 95% CI 0.0.84–1.33, *p* = 0.665). The ICU mortality of patients meeting the criteria for severe ARDS were not different between the cohorts (172/410, 42% vs. 48/107, 44.9%, respectively; RR 1.10, 95% CI 0.78–1.54, *p* = 0.661). 

### 3.1. ARDS Subsets at Baseline

At study entry, 792 patients (79.2%) from the testing cohort and 223 patients (73.6%) from the confirmatory cohort had PaO_2_/FiO_2_ < 150 mm Hg (Table 1). Their ICU mortality rate was not higher than that of patients with PaO_2_/FiO_2_ ≥ 150 mm Hg (testing cohort: 309/792 (39.0%) vs. 66/208 (31.7%); RR 1.22, 95% CI 0.0.99–1.53, *p* = 0.064; confirmatory cohort: 85/223 (38.1%) vs. 27/80 (33.8%); RR 1.13, 95% CI 0.80–1.60, *p* = 0.503). 

Almost one-third of the patients (423/1303, 32.5%) were on PEEP < 10 cm H_2_O (313/1000 (31.3%) in the testing cohort and 110/303 (36.3%) in the confirmatory cohort), and their ICU mortality rates were not different from those of patients at PEEP ≥ 10 cm H_2_O (testing cohort: 122/313 (39.0%) vs. 253/687 (36.8%); RR 1.06, 95% CI 0.89–1.1.25, *p* = 0.527; confirmatory cohort: 43/110 (39.1%) vs. 69/193 (35.8%); RR 1.09, 95% CI 0.81–1.48, *p* = 0.647). The ICU mortality rates were not different among the four subsets (*p* = 0.229 for the testing cohort and *p* = 0.432 for the confirmatory cohort) (Table 2, Figure S1A). 

### 3.2. ARDS Subsets at 24 h after Moderate/Severe ARDS Diagnosis

**Figure 1:**
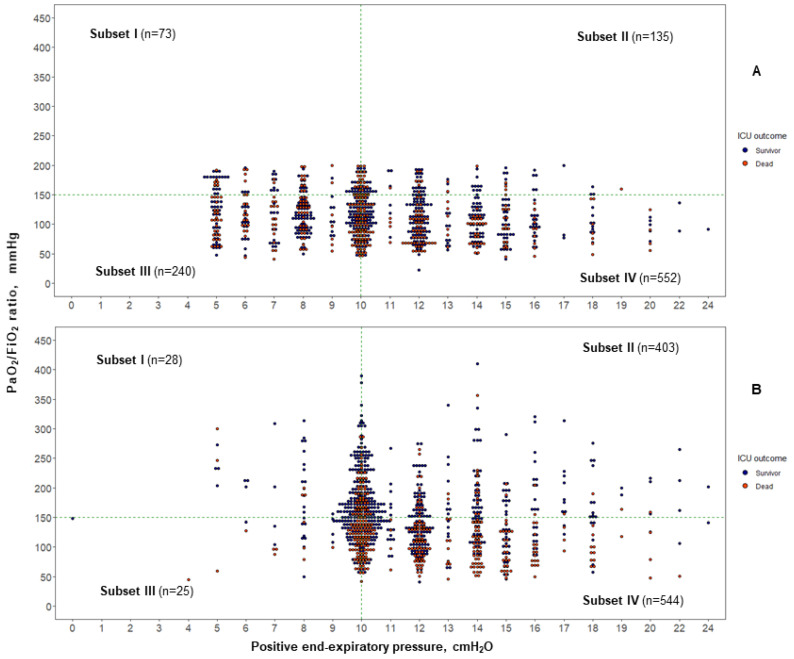
Distribution of 1000 patients (testing cohort) with moderate to severe acute respiratory distress syndrome (ARDS), based on cutoff values for the PaO_2_/FiO_2_ ratio (150 mm Hg) and the positive end-expiratory pressure level (10 cm H_2_O) for each individual patient: (**A**) at the time of moderate/severe ARDS diagnosis (baseline); (**B**) after 24 h of standard critical care with protective mechanical ventilation. The dotted lines are placed at a PaO_2_/FiO_2_ ratio of 150 mm Hg and at PEEP 10 cm H_2_O. Mortality increased as lung function deteriorated (from Subset I to Subset IV) at 24 h. Subset I: PaO_2_/FiO_2_ ≥ 150 at PEEP < 10; Subset II: PaO_2_/FiO_2_ ≥ 150 at PEEP ≥ 10; Subset III: PaO_2_/FiO_2_ < 150 at PEEP < 10; Subset IV: PaO_2_/FiO_2_ < 150 at PEEP ≥ 10.

Only five patients (four in the testing and one in the confirmatory cohorts) died before 24 h after enrollment. Since these five patients had PaO_2_/FiO_2_ ≤ 100 mm Hg at PEEP ≥ 10 cm H_2_O and FiO_2_ > 0.5, they were included in the 24-h analysis. The distribution of the patients in each subset changed markedly at 24 h (Table 2, Figure 1 and Figure S1B). In total, 569 patients (56.9%) in the testing cohort and 136 patients (44.9%) in the confirmatory cohort still had a PaO_2_/FiO_2_ < 150 mm Hg, and their ICU mortality rate was much higher than that of patients with PaO_2_/FiO_2_ ≥ 150 (278/569 (48.9%) vs. 97/431 (22%); RR 2.17, 95% CI 1.79–2.64, *p* < 0.0001 in the testing cohort; and 80/136 (58.8%) vs. 32/167 (19.2%); RR 3.07, 95% CI 2.18–4.32, *p* < 0.0001 in the confirmatory cohort). The classification into four subsets at 24 h of ARDS diagnosis showed a strong association with ICU mortality (*p* < 0.001) (Figure 1B and Figure S1B). Cross-validation of the pooled data of the testing cohort confirmed that each individual study validated the subset model (Table S1). 

At 24 h, patients in Subset IV showed higher mean SOFA scores than patients in the other subsets (Table 3 and Table S2). In Subset I, only five patients (17.9 %) from the testing cohort and five patients (14.3%) from the confirmatory cohort died in the ICU. The causes of death for each subset are listed in Table S3.

### 3.3. Probability of ICU Survival to Day 30

When considering the combined population of 1303 patients, patients with PaO_2_/FiO_2_ ≥ 150 mm Hg (Subsets I and II) had higher 30-day cumulative ICU survival compared with patients with PaO_2_/FiO_2_ < 150 mm Hg (Subsets III and IV) (Figure S2). Since Subsets I and III at 24 h of ARDS diagnosis had small sample sizes (*n* = 56 and *n* = 39, respectively), and a low number of ICU deaths (*n* = 9 and *n* = 17, respectively), they were of little interest in this study for predicting ICU mortality and did not influence the outcome or a clinical understanding. As a result, we aggregated patients with PaO_2_/FiO_2_ ≥ 150 mm Hg (Subsets I and II) and patients with PaO_2_/FiO_2_ < 150 mm Hg (Subsets III and IV) for examining the overall 30-day ICU cumulative survival in the two categories. 

**Figure 2:**
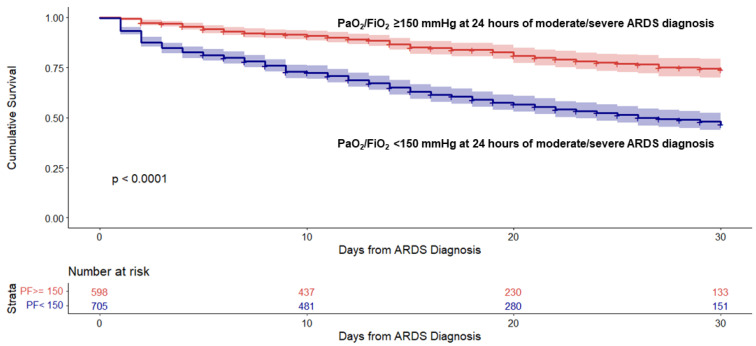
Probability of cumulative ICU survival to Day 30 in 1303 patients with moderate to severe acute respiratory distress syndrome (ARDS). Patients were stratified into two large categories based on cutoff values of 150 mm Hg for PaO_2_/FiO_2_ (<150 and ≥150) at 24 h of study entry.

Patients with PaO_2_/FiO_2_ ≥ 150 mm Hg at 24 h had 30-day higher survival compared with patients with PaO_2_/FiO_2_ < 150 mm Hg (HR 2.8, 95% CI 2.2–3.5, *p* < 0.0001) (Table S4, Figure 2). In general, patients with PaO_2_/FiO_2_ < 150 mm Hg at 24 h had a higher mean plateau pressure, required higher levels of FiO_2_, had a higher SOFA score, and had fewer ventilator-free days (Table S4).

When the impact of PaO_2_/FiO_2_ < 150 mm Hg on ICU mortality was adjusted by the patients’ age and SOFA scores, PaO_2_/FiO_2_ < 150 at 24 h remained the major determinant of ICU death (OR 3.1 (95% CI 2.4–4.0) (Table S5).

### 3.4. Additional Analysis with Different PaO_2_/FiO_2_ Cutoff Values

PaO_2_/FiO_2_ cutoff values of 100 or 120 mm Hg did not provide more reliable outcome predictions at ARDS diagnosis or at 24 h after diagnosis (Tables S6 and S7).

## 4. Discussion

The major findings of this study are as follows. First stratification of moderate to severe ARDS patients at onset/diagnosis based on PaO_2_/FiO_2_ ratio greater than vs. less than 150 mm Hg did not predict ICU mortality. Second, the two major categories of patients based on PaO_2_/FiO_2_ at 24 h had markedly different ICU outcomes: for PaO_2_/FiO_2_ ≥ 150 mm Hg, the ICU mortality rate was about 20% and for PaO_2_/FiO_2_ < 150 mm Hg, the ICU mortality rate was greater than 45%. 

The baseline gas exchange criteria for the definition of ARDS captured a highly heterogeneous group of patients with a spectrum of severity that represented fundamental differences in their pathophysiology and responses to specific therapies [38]. By changing the time of reassessment of the oxygenation defect to 24 h, and by using standardized ventilator settings, we identified subsets of moderate/severe ARDS patients with markedly different levels of risk of ICU death. Since there is no typical ARDS patient [1], the most critical factor in managing ARDS is the initiation of lung-protective MV after intubation [6], although the optimal MV strategy remains uncertain. The history of interventional RCTs of ARDS is full of failures, with few successes in the last decade [39]. Most RCTs of ARDS have not tested whether the experimental management or therapy is beneficial after assessing the degree of hypoxemia after 24 h of routine intensive management prior to randomization [39,40]. We need an ARDS classification system that can serve as a prototype to help set individual therapeutic targets, as in other critical conditions [41]. Developing an ARDS-specific stratification or sub-phenotyping model for guiding therapy and predicting outcomes is clinically relevant, because this heterogeneous syndrome is complex and evolves rapidly, and more than one-third of patients with moderate to severe ARDS do not leave the hospital alive.

Although categorization of continuous predictors (such as below vs. above a certain cutoff) should be avoided in the development of a model, the exception is when a well-accepted threshold is used in clinical practice [42]. A PaO_2_/FiO_2_ cutoff of 150 mm Hg seems to be appropriate, not only to discriminate between patients with higher or lower mortality but also for guiding medical therapy [18,19,20,21,22,23,24,25,26,43]. In our study, almost one-third of patients (423/1303, 32.5%) were ventilated with PEEP < 10 cm H_2_O at the time of moderate/severe ARDS diagnosis, a finding that is in line with recent reports on most successful and unsuccessful RCTs conducted in ARDS patients and published since 2010, where many patients were ventilated with PEEP < 10 cm H_2_O on average at inclusion into the trial and on the first day of randomization [39,44]. The European Collaborative Study [19] performed an observational study from 1985 to 1987 and analyzed 583 patients with ARDS in which hypoxemia was defined as a PaO_2_ < 75 mm Hg with FiO_2_ ≥ 0.5 at PEEP ≥ 5 cm H_2_O for at least 24 h. In that study, the mortality rate of ARDS patients with PaO_2_/FiO_2_ < 150 at 24 h was almost double the mortality rate of patients with PaO_2_/FiO_2_ ≥ 150 mm Hg. Using a similar study design to the European Collaborative Study, Villar et al. [20] published a pilot study in 1999 in a small population of 56 patients meeting the American–European Consensus Conference definition for ARDS, and found that the responses of PaO_2_ to PEEP after 24 h of the meeting ARDS criteria allowed a clear separation of the patients into two different groups with markedly different mortality rates. Three recent RCTs used a value of PaO_2_/FiO_2_ < 150 mm Hg at PEEP ≥ 5 [21,22] or ≥8 cm H_2_O [24] to enroll patients during the first 24–48 h of ARDS diagnosis. It is plausible that in a substantial proportion of the patients in recent RCTs, the severity of lung injury was modest. If patients have a low risk of the condition to be prevented, any trial will not validate the value of the intervention in the study [45]. Optimizing the selection of ARDS patients is central to the likelihood of successful trial design.

Our classification system uses two variables, PaO_2_/FiO_2_ and PEEP, which are particularly relevant for the diagnosis and ventilatory management of ARDS. However, we think that the key feature in our study was the time (waiting for 24 h), while PEEP was relatively unimportant because very few patients had PEEP < 10 cm H_2_O at 24 h after a diagnosis of moderate/severe ARDS. These ARDS subsets could be relevant for establishing prognoses, for selecting individualized therapies, and for helping to identify patients in whom benefit from treatment may be limited or disproportional to the resources used. Clearly, selection of the therapy for an individual ARDS patient involves both an assessment of respiratory dysfunction, as measured by the PaO_2_/FiO_2_ after 24 h of routine care, and an evaluation of the PEEP response. Not all ARDS is created equal. Distinguishing the level of lung severity of ARDS is critical for successful treatment, since there are certain ventilator and oxygenation therapeutic modalities that are not required in all patients. Subset I represents the less severe ARDS patients. Although, ideally, one aim of ventilating ARDS patients is to recruit consolidated and atelectatic alveolar units and decrease ventilator-induced lung injury [6], less than 50% of patients in our study achieved a PaO_2_/FiO_2_ ≥ 150 mm Hg with PEEP ≥ 10 at 24 h. It is plausible that the mortality rates in Subsets I and II are so low that it may have reached a lower limit that was dictated more by the underlying disease than by the syndrome itself [46]. Although a diagnosis of ARDS does not suggest any specific pharmacologic treatment, the underlying conditions or etiological diseases that cause ARDS are important root causes of mortality in ARDS [27]. The enrollment of mechanically ventilated ARDS patients with rapidly improving ARDS, such as those in Subsets I and II, may contribute to the failure of therapeutic RCTs [47]. Some patients from Subset III may have had a PaO_2_/FiO_2_ < 150 mm Hg as a result of insufficient PEEP, although in some patients, the best PEEP, according to lung compliance, could be <10 cm H_2_O [15,48]. Patients in Subset IV represent the most critical category and appear to be very resistant to therapy, suggesting that these patients should be the target for aggressive and innovative therapies. Further validations and evaluations of interventions for each subset are necessary. 

Our study has several strengths. First, we have studied a large population of patients with moderate to severe ARDS managed with lung-protective MV admitted to a multidisciplinary network of ICUs. Since this was an observational study with practically no exclusion criteria, we believe that our patient population represents unselected patients under the wide syndromic umbrella of moderate to severe ARDS. We do not think that there was a relevant effect of time on our findings, since this type of combined analysis of several hundreds of patients from independent cohorts has been used extensively by other authors using heterogeneous populations from previous published clinical trials [49,50,51] (see the Supplementary File). Our model applies only to patients with moderate/severe ARDS while they are intubated and mechanically ventilated in line with a lung-protective ventilation approach. Second, our classification system is in line with recent recommendations [52] stating that better identification of patient populations is the key for appropriate characterization of the patients’ status. Third, PaO_2_/FiO_2_ was examined in line with a standardized ventilatory approach at 24 h, although five severe ARDS patients died before 24 h, and 20 patients with PaO_2_/FiO_2_ < 150 mm Hg were not assessed at PEEP ≥ 10 cm H_2_O for several reasons (see the Supplementary File). 

We also acknowledge that our study has potential limitations. First, we did not enroll ARDS patients with persistent PaO_2_/FiO_2_ > 200 mm Hg during the ICU stay (see the Supplementary File). However, we do not believe that the exclusion of “mild” ARDS weakens our findings, since patients with mild ARDS represent a case-mix of patients in which many patients may not require invasive MV. Second, the classification at the time of moderate/severe ARDS onset/diagnosis, as mandated by the current ARDS definition, was not assessed using standardized ventilator settings, such as we applied at 24 h. However, a previous report examining the baseline PaO_2_/FiO_2_ values under standardized ventilatory settings showed that the baseline PaO_2_/FiO_2_ was not helpful for predicting the outcome compared with PaO_2_/FiO_2_ at 24 h [7,9]. Third, regarding the limitation of waiting 24 h for enrolling patients into RCTs, we need other studies to examine whether these ARDS subsets could be established at 12 or 18 h after ARDS diagnosis. Although most RCTs in ARDS since 1990 have considered enrolling patients within 24–48 h after ARDS diagnosis [44], in some trials, patients were enrolled at a median or mean time of 7.6 h [24], 22 h [21], or 33 h [22], although others included patients enrolled at <72 h from ARDS diagnosis [53]. Fourth, similar to most clinical investigators, we did not assess the compliance of physicians with our recommended guidelines for many therapies. Finally, we could address the impact that the Spanish healthcare system may have had on the generalizability of our results to other healthcare systems.

In conclusion, two clinical variables, namely PaO_2_/FiO_2_ (<150 mm Hg vs. ≥150 mm Hg) and PEEP (<10 cm H_2_O vs. ≥10 cm H_2_O) at 24 h after moderate/severe ARDS onset, seem to be essential for identifying ARDS subsets that could be used to guide medical therapy, to predict ICU outcomes, and to enroll patients in RCTs. Further confirmation and impact studies should evaluate whether the implementation of this classification and prediction model in clinical practice improves patient outcomes by informing therapeutic decisions. 

## Figures and Tables

**Table 1:** Baseline characteristics and outcome data of 1303 patients with moderate to severe acute respiratory distress syndrome (ARDS).

Variables	Testing Cohort (*n* = 1000)	Confirmatory Cohort (*n* = 303)
Age, years, mean (SD)	57 ± 16	58 ± 15
Sex	*n* (%)	*n* (%)
Male	680 (68.0)	223 (73.6)
Female	320 (32.0)	80 (26.4)
Etiology	*n* (%)	*n* (%)
Pneumonia	480 (48.0)	110 (36.3)
Sepsis	286 (28.6)	78 (25.7)
Aspiration	94 (9.4)	47 (15.5)
Trauma	74 (7.4)	38 (12.5)
Acute pancreatitis	32 (3.2)	13 (4.3)
Multiple transfusions	10 (1.0)	3 (1.0)
Others	24 (2.4)	14 (4.6)
Degree of ARDS severity	*n* (%)	*n* (%)
Severe	410 (41.0)	107 (35.3)
Moderate	590 (59.0)	196 (64.7)
APACHE II score, mean ± SD	20.8 ± 6.7	21.3 ± 7.8 ^¶^
SOFA score, mean ± SD	9.1 ± 3.5	9.8 ± 3.5
PaO_2_/FiO_2_, mm Hg, mean ± SD	114.3 ± 38.4	120.4 ± 41.0
FiO_2_, mean ± SD	0.79 ± 0.19	0.76 ± 0.20
PaO_2_, mm Hg, mean ± SD	85.9 ± 26.3	86.3 ± 24.9
PaCO_2_, mm Hg, mean ± SD	49.0 ±12.5	50.6 ± 13.8
pH, mean ± SD	7.30 ± 0.11	7.29 ± 0.11
VT, mL/kg PBW, mean ± SD	6.8 ± 1.0	6.7 ± 1.1
Respiratory rate, resp/min, mean ± SD	21.3 ± 4.9	22.3 ± 4.6
Minute ventilation, L/min, mean ± SD	9.1 ± 2.2	9.5 ± 2.0
PEEP, cm H_2_O, mean ± SD	12 ± 3	11 ± 3
Plateau pressure, cm H_2_O, mean ± SD	26.5 ± 4.8 ^§^	25.2 ± 4.9
Driving pressure, cm H_2_O, mean ± SD	14.5 ± 4.8 ^§^	14.3 ± 4.8
No. extrapulmonary OF, mean ± SD	1.7 ± 1.1	1.8 ± 1.1
Length of ICU stay, d, median (P_25_–P_75_)	19 (11–31)	16 (9–27)
Duration of MV from ARDS diagnosis, d, mean ± SD	17.6 ± 17.0	14.0 ± 16.6
VFDs, d, mean ± SD	7.9 ± 9.1	9.2 ± 9.7
Days from ICU admission to ARDS onset, median (P_25_–P_75_)	1 (0–3)	1 (0–2)
Days from ARDS onset to ICU discharge, median (P_25_–P_75_)	16 (9–28)	14 (7–23)
All-cause ICU mortality, n (%)	375 (37.5)	112 (37.0)
All-cause hospital mortality, n (%)	415 (41.5)	124 (40.9)

APACHE: Acute Physiology and Chronic Health Evaluation; d: days; FiO_2_: fraction of inspired oxygen concentration; ICU: intensive care unit; MV: mechanical ventilation; OF: organ failure; PBW: predicted body weight; PEEP: positive end-expiratory pressure; SD: standard deviation; SOFA: sequential organ failure assessment scale; VFDs: ventilator-free days from the diagnosis of moderate/severe ARDS until Day 28; VT: tidal volume. ^¶^ The APACHE II score was not reported at baseline in 19 patients. ^§^ Plateau pressure was not reported at baseline in 15 patients.

**Table 2:** Distribution and mortality in the intensive care unit (ICU) of each subset of patients with moderate to severe acute respiratory distress syndrome (ARDS) in the testing (*n* = 1000) and confirmatory (*n* = 303) cohorts.

Cohort	Timing	Subset I PaO_2_/FiO_2_ ≥ 150 at PEEP < 10	Subset II PaO_2_/FiO_2_ ≥ 150 at PEEP ≥ 10	Subset III PaO_2_/FiO_2_ < 150 at PEEP < 10	Subset IV PaO_2_/FiO_2_ < 150 at PEEP ≥ 10	*p*-Value
**Testing Cohort**	**At moderate/severe ARDS diagnosis**					
	No. of subjects	73	135	240	552	
	No. events (ICU deaths)	25	41	97	212	
	Event rate (95% CI)	34.3(23.4–45.1)	30.4(22.6–38.1)	40.4(34.2–46.6)	38.4(34.4–42.5)	0.184
	Risk ratio (95% CI)	1 (Ref)	0.9 (0.6–1.3)	1.2 (0.8–1.7)	1.1 (0.8–1.6)	0.229
	**At 24 h after onset**					
	No. of subjects	28	403	25	544	
	No. events (ICU deaths)	5	92	10	268	
	Event rate (95% CI)	17.9 (3.7–32.0)	22.8(18.7–26.9)	40.0(20.8–59.2)	49.3(45.0–53.6)	<0.001
	Risk ratio (95% CI)	1 (Ref)	1.6 (0.6–2.9)	2.2 (0.9–5.7)	2.8 (1.2–6.1)	<0.001
**Confirmatory Cohort**	**At moderate/severe ARDS diagnosis**					
	No. of subjects	32	48	78	145	
	No. events (ICU deaths)	9	18	34	51	
	Event rate (95% CI)	28.1(12.6–43.7)	37.5(24.0–52.7)	43.6(32.4–55.3)	35.2(27.4–42.9)	0.745
	Risk ratio (95% CI)	1 (Ref)	1.3 (0.7–2.6)	1.6 (0.8–2.8)	1.3 (0.7–2.3)	0.434
	**At 24 h after onset**					
	No. of subjects	28	139	14	122	
	No. events (ICU deaths)	4	28	7	73	
	Event rate (95% CI)	14.3 (1.3–27.3)	20.1(13.5–26.8)	50.0(23.8–76.2)	59.8(51.1–68.5)	<0.001
	Risk ratio (95% CI)	1 (Ref)	1.4 (0.5–3.7)	3.5 (1.2–10.0)	4.2 (1.7–10.5)	<0.001

ARDS: acute respiratory distress syndrome; ICU: intensive care unit; PEEP: positive end-expiratory pressure.

**Table 3:** Main characteristics of 1000 patients with moderate to severe acute respiratory distress syndrome (ARDS). The classification was made at 24 h after diagnosis of moderate/severe ARDS as Subsets I, II, III, and IV based on cutoff values of 150 mm Hg for PaO_2_/FiO_2_ and 10 cm H_2_O for PEEP *.

Variables	Values				
	Subset I *n* = 28	Subset II *n* = 403	Subset III *n* = 25	Subset IV *n* = 544	*p*-Value
APACHE II ^¶^					
Mean ± SD	16.4 ± 4.2	17.5 ± 7.2	20.1 ± 6.4	20.4 ± 7.0	<0.001
Mean difference (95% CI)	0 (Ref)	1.1 (−1.6 to 3.8)	3.7 (0.7 to 6.7)	4.0 (1.4 to 6.6)	<0.001
Age, mean ± SD	66 ± 13	56 ± 16	60 ± 19	57 ± 16	0.011
Sex, No. (%)					0.046
Men	15 (53.6)	263 (65.3)	21 (84.0)	381 (70.0)	
Women	13 (46.4)	140 (34.7)	4 (16.0)	163 (30.0)	
VT, mL/kg PBW					
Mean ± SD	6.8 ± 0.9	6.7 ± 0.9	6.7 ± 0.8	6.6 ± 0.9	0.285
Mean difference (95% CI)	0 (Ref)	−0.1 (−0.4 to 0.2)	−0.1 (−0.6 to 0.4)	−0.2 (−0.5 to 0.1)	0.252
Plateau pressure, cm H_2_O					
Mean ± SD	24.4 ± 5.0	25.2 ± 4.6	26.2 ± 4.6	28.0 ± 4.3	<0.001
Mean difference (95% CI)	0 (Ref)	0.8 (−1.0 to 2.6)	1.8 (−0.9 to 4.5)	3.6 (2.0 to 5.3)	<0.001
PEEP, cm H_2_O					
Mean ± SD	7.6 ± 1.7	12.5 ± 2.8	7.4 ± 2.1	13.0 ± 2.8	<0.001
Mean difference (95% CI)	0 (Ref)	4.9 (3.8 to 6.0)	−0.2 (−1.2 to 0.8)	5.4 (4.4 to 6.5)	<0.001
Driving pressure, cm H_2_O					
Mean ± SD	16 ± 5	12 ± 4	18 ± 5	15 ± 4	<0.001
Mean difference (95% CI)	0 (Ref)	−4 (−6 to −3)	2 (−1 to 5)	−1 (−2 to 1)	<0.001
FiO_2_					
Mean ± SD	0.53 ± 0.11	0.55 ± 0.11	0.77 ± 0.16	0.75 ± 0.18	<0.001
Mean difference (95% CI)	0 (Ref)	0.02(−0.1 to 0.1)	0.24 (0.2 to 0.7)	0.22 (0.1 to 0.3)	<0.001
PaO_2_/FiO_2_, mm Hg					
Mean ± SD	228 ± 45	200 ± 46	110 ± 30	107 ± 27	<0.001
Mean difference (95% CI)	0 (Ref)	−28 (−46 to −10)	−118(−139 to −97)	−121(−132 to −110)	<0.001
SOFA score					
Mean ± SD	7.3 ± 3.5	8.1 ± 3.3	8.2 ± 3.3	9.9 ±3.8	<0.001
Mean difference (95% CI)	0 (Ref)	0.8 (−0.5 to 2.1)	0.9 (−1.0 to 2.8)	2.6 (1.2 to 4.0)	<0.001
Days on MV from ARDS diagnosis					
Mean ± SD	12.3 ± 13.1	16.2 ± 14.9	16.3 ± 14.3	19.0 ± 18.5	0.025
Mean difference (95% CI)	0 (Ref)	3.9 (−1.8 to 9.6)	4.0(−3.6 to 11.6)	6.7(−0.3 to 13.7)	0.059
VFDs, d					
Mean ± SD	13.7 ± 11.	11.1 ± 9.5	6.8 ± 8.8	5.2 ± 7.8	<0.001
Mean difference (95% CI)	0 (Ref)	−2.6 (−6.3 to 1.1)	−6.9(−12.4 to −1.4)	−8.5(−11.5 to −5.5)	<0.001
ICU deaths					
No. events	5	92	10	268	
Event rate (95% CI)	17.9 (3.7–32.0)	22.8 (18.7–26.9)	40.0 (20.8–59.2)	49.3 (45.1–53.5)	<0.001
Risk ratio (95% CI)	1 (Ref)	1.3 (0.6–2.9)	2.2 (0.9–5.7)	2.8 (1.2–6.1)	<0.001

APACHE: Acute Physiology and Chronic Health Evaluation; ARDS: acute respiratory distress syndrome; ICU: intensive care unit; MV: mechanical ventilation; PBW: predicted body weight; PEEP: positive end-expiratory pressure; SD: standard deviation; SOFA: sequential organ failure assessment; VFDs: ventilator-free days from diagnosis of moderate/severe ARDS until Day 28; VT: tidal volume. ^¶^ The APACHE II score was not reported at 24 h in 33 patients (10 patients in Subset II, 2 patients in Subset III, and 21 in Subset IV). * Subset I, PaO_2_/FiO_2_ ≥ 150 at PEEP < 10; Subset II, PaO_2_/FiO_2_ ≥ 150 at PEEP ≥ 10; Subset III, PaO_2_/FiO_2_ < 150 at PEEP < 10; Subset IV, PaO_2_/FiO_2_ < 150 at PEEP ≥ 10.

## Data Availability

All data needed to evaluate the conclusions in this article are presented and tabulated in the main text or the Supplementary File. Data are available from the corresponding author on reasonable request.

## Supplementary Materials

The following supporting information can be downloaded at: https://www.mdpi.com/article/10.3390/jcm11195724/s1.

## Abbreviations

APACHE II: Acute Physiology and Chronic Health Evaluation II score; ARDS: acute respiratory distress syndrome; CI: confidence intervals; FiO_2_: fraction of inspired oxygen; HR: hazard ratio; ICU: intensive care unit; MV: mechanical ventilation; OR: odds ratio; PaCO_2_: partial pressure of carbon dioxide in arterial blood; PaO_2_: partial pressure of arterial oxygen; PaO_2_/FiO_2_: partial pressure of oxygen in arterial blood/inspired oxygen fraction ratio; PBW: predicted body weight; PEEP: positive end-expiratory pressure; P_25_–P_75_: 25th–75th percentiles; RCT: randomized controlled trial; RR: risk ratio; SD: standard deviation; SIESTA: Spanish Initiative for Epidemiology, Stratification, and Therapies of ARDS; SOFA: Sequential Organ Failure Assessment; SpO_2_: oxygen saturation; STROBE: Strengthening the Reporting of Observational Studies in Epidemiology; VFD: ventilator-free days; VT: tidal volume.

## Appendix A. Members of the SIESTA Network Are Listed below

- Jesús Villar, Rosa L. Fernández, Cristina Fernández, Jesús M. González-Martin, Pedro Rodríguez-Suárez (Hospital Universitario Dr. Negrín, Las Palmas de Gran Canaria, Spain);
- Alfonso Ambrós, Rafael del Campo, Carmen Martín-Rodríguez, Ana Bueno-González, Carmen Hornos-López (Hospital General Universitario, Ciudad Real, Spain);
- Fernando Mosteiro, Ana M. Díaz-Lamas, Regina Arrojo, Lidia Pita-García (Complejo Hospitalario Universitario de La Coruña, La Coruña, Spain);
- Lorena Fernández, Jesús Sánchez-Ballesteros, Jesús Blanco, Arturo Muriel, Pablo Blanco-Schweizer, José Ángel de Ayala, César Aldecoa, Jesús Rico-Feijoo, Alba Pérez, Silvia Martín-Alfonso (Hospital Universitario Río Hortega, Valladolid, Spain);
- Domingo Martínez, Juan A. Soler, Ana M. del Saz-Ortiz, Luís A. Conesa-Cayuela (Hospital Universitario Virgen de Arrixaca, Murcia, Spain);
- Demetrio Carriedo, Ana M. Domínguez-Berrot, Francisco J. Díaz-Domínguez, Raúl I. González-Luengo (Complejo Hospitalario Universitario de León, León, Spain);
- Lucia Capilla (Hospital General Universitario Rafael Méndez, Lorca, Murcia, Spain);
- David Andaluz, Leonor Nogales, Laura Parra (Hospital Clínico Universitario, Valladolid, Spain);
- Elena González-Higueras, Rosario Solano, María J. Bruscas (Hospital Virgen de la Luz, Cuenca, Spain);
- Blanca Arocas, Marina Soro, Javier Belda, Andrea Gutiérrez, Ernesto Pastor, Gerardo Aguilar (Hospital Clínico Universitario, Valencia, Spain);
- Carlos Ferrando (Hospital Clinic, Barcelona, Spain);
- José M. Añón, Belén Civantos, Mónica Hernández (Hospital Universitario La Paz, Madrid, Spain);
- Raquel Montiel, Dácil Parrilla, Eduardo Peinado, Lina Pérez-Méndez (Hospital Universitario NS de Candelaria, Tenerife, Spain);
- Anxela Vidal, Denis Robaglia, César Pérez (Hospital Universitario Fundación Jiménez Díaz, Madrid, Spain);
- María del Mar Fernández (Hospital Universitario Mutua Terrassa, Terrassa, Barcelona, Spain);
- Eleuterio Merayo, Chanel Martínez-Jiménez, Ángeles de Celis-Álvarez (Hospital del Bierzo, Ponferrada, León, Spain);
- Juan M. Mora-Ordoñez, J. Francisco Martínez-Carmona, Álvaro Valverde-Monto, Victoria Olea-Jiménez (Hospital Regional Universitario de Málaga, Málaga, Spain);
- Concepción Tarancón, Silvia Cortés-Díaz (Hospital Virgen de la Concha, Zamora, Spain);
- Carmen Martín-Delgado (Hospital La Mancha Centro, Alcázar de San Juan, Ciudad Real, Spain);
- Francisca Prieto (Hospital Santa Bárbara, Puertollano, Ciudad Real, Spain);
- Isidro Prieto, Mario Chico, Darío Toral (Hospital Universitario 12 de Octubre, Madrid, Spain);
- Miguel A. Romera, Carlos Chamorro-Jambrina (Hospital Universitario Puerta de Hierro, Majadahonda, Madrid, Spain);
- Alec Tallet, Santiago Macías, Noelia Lázaro (Hospital General de Segovia, Segovia, Spain);
- Isabel Murcia, Ángel E. Pereyra (Hospital General Universitario de Albacete, Albacete, Spain);
- Francisco Alba, Ruth Corpas (Hospital NS del Prado, Talavera de la Reina, Toledo, Spain);
- David Pestaña, Pilar Cobeta, Adrián Mira (Hospital Universitario Ramón y Cajal, Madrid, Spain);
- Francisca Prieto (Hospital Santa Barbara, Puertollano, Ciudad Real, Spain);
- Lluis Blanch, Gemma Gomá, Gisela Pili (Corporació Sanitaria Parc Taulí, Sabadell, Barcelona, Spain);
- Antonio Santos-Bouza, Cristina Domínguez (Complejo Hospitalario Universitario de Santiago, Santiago de Compostela, La Coruña, Spain);
- Javier Collado, José I. Alonso (Hospital Río Carrión, Palencia, Spain);
- Alberto Indarte, María E. Perea (Hospital General Yagüe, Burgos, Spain);
- Ricardo Fernández, José I. Lozano (Hospital de Hellín, Albacete, Spain)
- Ewout W. Steyerberg (Department of Biomedical Data Sciences, Leiden University Medical Center, Leiden, The Netherlands);
- Robert M. Kacmarek (deceased), Lorenzo Berra (Massachussets General Hospital, Boston, Massachusetts, USA);
- Arthur S. Slutsky (Li Ka Shing Knowledge Institute, St. Michael’s Hospital, Toronto, Ontario, Canada).

## References

[1] Villar J. (2011). What is the acute respiratory distress syndrome?. Respir. Care.

[2] Ranieri V.M., Rubenfeld G.D., Thompson B.T., Ferguson N.D., Caldwell E., Fan E., Camporota L., Slutsky A.S. (2012). Acute respiratory distress syndrome: The Berlin definition. JAMA.

[3] Villar J., Pérez-Méndez L., Kacmarek R.M. (2016). The Berlin definition met our needs: No. Intensive Care Med..

[4] Peng J.M., Qian C.Y., Yu X.Y., Zhao M.Y., Li S.S., Ma X.C., Kang Y., Zhou F.C., He Z.H., Qin T.H. (2017). Does training improve diagnostic accuracy and inter-rater agreement in applying the Berlin radiographic definition of acute respiratory distress syndrome? A multicenter prospective study. Crit. Care.

[5] Fountain S., Assar S., Heise C., Curry S., Raschke R.A. (2018). What’s in a chest radiograph? Inter-rater variability in determining acute respiratory distress syndrome. Am. J. Resp. Crit. Care Med..

[6] Villar J., Slutsky A.S. (2017). GOLDEN anniversary of the acute respiratory distress syndrome: Still much work to do!. Curr. Opin. Crit. Care.

[7] Villar J., Blanco J., del Campo R., Andaluz-Ojeda D., Díaz-Domínguez F.J., Muriel A., Córcoles V., Suárez-Sipmann F., Tarancón C., González-Higueras E. (2015). Assessment of PaO_2_/FiO_2_ for stratification of patients with moderate and severe acute respiratory distress syndrome. BMJ Open.

[8] Hernu R., Wallet F., Thiolliére F., Martin O., Richard J.C., Schmitt Z., Wallon G., Delannoy B., Rimmelé T., Démeret C. (2013). An attempt to validate the modification of the American-European consensus definition of acute lung injury/acute respiratory distress syndrome by the Berlin definition in a university hospital. Intensive Care Med..

[9] Villar J., Pérez-Méndez L., Blanco J., Añón J.M., Blanch L., Belda J., Santos-Souza A., Fernández R.L., Kacmarek R.M., Spanish Initiative for Epidemiology, Stratification, and Therapies for ARDS (SIESTA) Network (2013). A universal definition of ARDS: The PaO_2_/FiO_2_ ratio under a standard ventilatory setting—A prospective, multicenter validation study. Intensive Care Med..

[10] Huber W., Findeissen M., Lahmer T., Herner A., Rasch S., Mayr U., Hoppmann P., Jaitner J., Okrojek R., Brettner F. (2020). Prediction of outcome in patients with ARDS: A prospective cohort study comparing ARDS-definitions and other ARDS-associated parameters, ratios and scores at intubation and over time. PLoS ONE.

[11] Phillips C.R. (2013). The Berlin definition: Real change or the emperor’s new clothes?. Crit. Care.

[12] Villar J., Pérez-Méndez L., López J., Belda J., Blanco J., Saralegui I., Suárez-Sipmann F., López J., Lubillo S., Kacmarek R.M. (2007). An early PEEP/FiO_2_ trial identifies different degrees of lung injury in patients with acute respiratory distress syndrome. Am. J. Respir. Crit. Care Med..

[13] Kamo T., Tasaka S., Susuki T., Asakura T., Susuki S., Yagi K., Namkoong H., Ishii M., Morisaki H., Betsuyaku T. (2019). Prognostic values of the Berlin definition criteria, blood lactate levels, and fibroproliferative changes on high-resolution computed tomography in ARDS patients. BMC Pulm. Med..

[14] Shapiro B.A., Cane R.D., Harrison R.A. (1984). Positive end-expiratory pressure therapy in adults with special reference to acute lung injury: A review of the literature and suggested clinical correlations. Crit. Care Med..

[15] Cove M.E., Pinsky M.R., Marini J.J. (2022). Are we ready to think differently about setting PEEP?. Crit. Care.

[16] Briel M., Meade M., Mercat A., Brower R.G., Talmor D., Walter S.D., Slutsky A.S., Pullenayegum E., Zhou Q., Cook D. (2010). Higher vs lower positive end-expiratory pressure in patients with acute lung injury and acute respiratory distress syndrome: Systematic review and meta-analysis. JAMA.

[17] Kang M., Kempker J.A. (2019). Definitions, epidemiology, clinical risk factors, and health disparities in acute respiratory distress syndrome. Sem. Respir. Crit. Care Med..

[18] Bone R.C., Maunder R., Slotman G., Silverman H., Hyers T.M., Kerstein M.D., Ursprung J.J. (1989). Prostaglandin E1 Study Group. An early test of survival in patients with the adult respiratory distress syndrome. The PaO2/FiO2 ratio and its differential response to conventional therapy. Chest.

[19] Artigas A., Carlet J., LeGall J.R., Chastang C., Blanch L., Fernández R., Zapol W.M., Lemaire F. (1991). Clinical presentation, prognostic factors and outcome of ARDS in the European Collaborative Study (1985–1987). Adult Respiratory Distress Syndrom.

[20] Villar J., Pérez-Méndez L., Kacmarek R.M. (1999). Current definitions of acute lung injury and the acute respiratory distress syndrome do not reflect their true severity and outcome. Intensive Care Med..

[21] Papazian L., Forel J.M., Gacouin A., Penot-Ragon C., Perrin G., Loundou A., Jaber S., Arnal J.M., Perez D., Seghboyan J.M. (2010). Neuromuscular blockers in early acute respiratory distress syndrome. N. Engl. J. Med..

[22] Guérin C., Reignier J., Richard J.C., Beuret P., Gacouin A., Boulain T., Clavel M., Chatellier D., Jaber S., Rosselli S. (2013). Prone positioning in severe acute respiratory distress syndrome. N. Engl. J. Med..

[23] Maiolo G., Collino F., Vasques F., Rapetti F., Tonetti T., Romitti F., Cressoni M., Chiumello D., Moerer O., Herrmann P. (2018). Reclassifying acute respiratory distress syndrome. Am. J. Respir. Crit. Care Med..

[24] Moss M., Huang D., Brower R.G., Ferguson N.D., Ginde A.A., Gong M.N., Grissom C.K., Gundel S., Hayden D., The National Heart, Lung, and Blood Institute Petal Clinical Trials Network (2019). Early neuromuscular blockade in the acute respiratpry distress syndrome. N. Engl. J. Med..

[25] Villar J., Fernández R.L., Ambrós A., Parra L., Blanco J., Domínguez-Berrot A.M., Gutiérrez J.M., Blanch L., Añón J.M., Martín C. (2015). A clinical classification of the acute respiratory distress syndrome for predicting outcome and guiding therapy. Crit. Care Med..

[26] Bos L.D., Cremer O.L., Ong D.S.Y., Caser E.B., Barbas C.S.V., Villar J., Kacmarek R.M., Schultz M.J., MARS Consortium (2015). External validation confirms the legitimacy of a new clinical classification of ARDS for predicting outcome. Intensive Care Med..

[27] Villar J., Martínez D., Mosteiro F., Ambrós A., Añón J.M., Ferrando C., Soler J.A., Montiel R., Vidal A., Conesa-Cayuela L.A. (2018). Stratification and Outcome of Acute Respiratory Distress Syndrome (STANDARDS) Network. Is overall mortality the right composite endpoint in clinical trials of acute respiratory distress syndrome?. Crit. Care Med..

[28] Villar J., Mora-Ordoñez J.M., Soler J.A., Mosteiro F., Vidal A., Ambrós A., Fernández L., Murcia I., Civantos B., Romera M.A. (2022). The PANDORA study: Prevalence and outcome of acute hypoxemic respiratory failure in the pre-COVID era. Crit. Care Explor..

[29] Von Elm E., Altman D.G., Egger M., Pocock S.J., Gøtzsche P.C., Vandenbroucke J.P., for the STROBE Initiative (2007). The strengthening the reporting of observational studies in epidemiology (STROBE) statement: Guidelines for reporting observational studies. PLoS Med..

[30] Vergouwe Y., Steyerberg E.W., Eijkemans M.J.C., Habbema J.D.F. (2005). Substantial effective sample sizes were required for external validation studies of predictive logistic regression models. J. Clin. Epidemiol..

[31] Knaus W.A., Draper E.A., Wagner D.P., Zimmerman J.E. (1985). APACHE II: A severity of disease classification system. Crit. Care Med..

[32] Vincent J.L., de Mendonça A., Cantraine F., Moreno R., Takala J., Suter P.M., Sprung C.L., Colardyn F., Blecher S. (1998). Use of the SOFA score to assess the incidence of organ dysfunction/failure in intensive care units: Results of a multicenter, prospective study. Working group on “sepsis-related problems” of the European Society of Intensive Care Medicine. Cri. Care Med..

[33] Acute Respiratory Distress Syndrome Network (2000). Ventilation with lower tidal volumes as compared with traditional tidal volumes for acute lung injury and the acute respiratory distress syndrome. N. Engl. J. Med..

[34] Fan E., Del Sorbo L., Goligher E.C., Hodgson C.L., Munshi L., Walkey A.J., Adhikari N.K.J., Amato M.B.P., Branson R., Brower R.G. (2017). American Thoracic Society, European Society of Intensive Care Medicine, and Society of Critical Care Medicine. An official American Thoracic Society/European Society of Intensive Care Medicine/Society of Critical Care Medicine Clinical Practice Guideline: Mechanical ventilation in adult patients with acute respiratory distress syndrome. Am. J. Respir. Crit. Care Med..

[35] Ioannidis J.P.A. (2019). The importance of predefined rules and prespecified statistical analyses. Do not abandon significance. JAMA.

[36] Villar J., Ambrós A., Mosteiro F., Martínez D., Fernández L., Ferrando C., Carriedo D., Soler J.A., Parrilla D., Hernández M. (2019). A prognostic enrichment strategy for selection of patients with acute respiratory distress syndrome in clinical trials. Crit. Care Med..

[37] Ioannidis J.P.A. (2018). The proposal to lower *p* value thresholds to 0.005. JAMA.

[38] Matthay M.A., McAuley D.F., Ware L.B. (2017). Clinical trials in acute respiratory distress syndrome: Challenges and opportunities. Lancet Respir. Med..

[39] Villar J., Ferrando C., Tusman G., Berra L., Rodríguez-Suárez P., Suárez-Sipmann F. (2021). Unsuccessful and successful clinical trials in acute respiratory distress syndrome: Addressing physiology-based gaps. Front. Physiol.

[40] Juschten J., Tuinman P.R., Guo T., Juffermans N.P., Schultz M.J., Loer S.A., Girbes A.R.J., de Grooth H.J. (2021). Between-trial heterogeneity in ARDS research. Intensive Care Med..

[41] Forrester J.S., Diamond G.A., Swan H.J.C. (1977). Correlative classification of clinical and hemodynamic function after acute myocardial infarction. Am. J. Cardiol..

[42] Steyerberg E.W., Vergouwe Y. (2014). Towards better clinical prediction models: Seven steps for development and an ABCD for validation. Eur. Heart J..

[43] Qadir N., Bartz R.R., Cooter M.L., Hough C.L., Lanspa M.J., Banner-Goodspeed V.M., Chen J.T., Giovanni S., Gomaa D., Sjoding M.W. (2021). Variation in early management practices in moderate-to-severe ADS in the United States. The Severe ARDS-generating evidence study. Chest.

[44] Villar J., Suárez-Sipmann F., Kacmarek R.M. (2017). Should the ART trial change our practice?. J. Thorac. Dis..

[45] Villar J., Pérez-Méndez L., Aguirre-Jaime A., Kacmarek R.M. (2005). Why are physicians so skeptical about positive randomized controlled trials in critical care medicine?. Intensive Care Med..

[46] Bernard G. (2017). Acute lung failure—our evolving understanding of ARDS. N. Engl. J. Med..

[47] Schenck E.J., Oromendia C., Torres L.K., Berlin D.A., Choi A.M.K., Siempos I.I. (2019). Rapidly improving ARDS in therapeutic randomized controlled trials. Chest.

[48] Rezoagli E., Bellani G. (2019). How I set up positive end-expiratory pressure: Evidence- and physiology-based. Crit. Care.

[49] Amato M.B., Meade M.O., Slutsky A.S., Brochard L., Costa E.L., Schoenfeld D.A., Stewerst T.E., Briel M., Talmor D., Mercat A. (2015). Driving pressure and survival in the acute respiratory distress syndrome. N. Engl. J. Med..

[50] Metkus T.S., Guallar E., Sokoll L., Morrow D., Tomaselli G., Brower R., Schulman S., Korley F.K. (2017). Prevalence and prognostic association of circulating troponin in acute respiratory distress syndrome. Crit. Care Med..

[51] Zhang R., Wang Z., Tejera P., Frank A.J., Wei Y., Su L., Zhu Z., Guo Y., Chen F., Bajwa E.K. (2017). Late-onset moderate to severe acute respiratory distress syndrome is associated with shorter survival and higher mortality: A two-stage association study. Intensive Care Med..

[52] Vincent J.L., Hall J.B., Slutsky A.S. (2015). Ten big mistakes in intensive care medicine. Intensive Care Med..

[53] Cavalcanti A.B., Suzumura E.A., Laranjeira L.N., Paisani D.M., Damiani L.P., Guimarães H.P., Romano E.R., Regenga M.M., Taniguchi L.N.T., Writing Group for the Alveolar Recruitment for Acute Respiratory Distress Syndrome Trial (ART) Investigators (2017). Effect of lung recruitment and titrated positive end-expiratory pressure (PEEP) vs low PEEP on mortality in patients with acute respiratory distress syndrome: A randomized clinical trial. JAMA.

