# Supplementary material for: Respiratory Subsets in Patients with Moderate to Severe Acute Respiratory Distress Syndrome for Early Prediction of Death
Source: J Clin Med. 2022 Sep 27;11(19):5724. doi: 10.3390/jcm11195724 (PMC9570540; doi:10.3390/jcm11195724)
Supplement: Supplementary file 1 [file jcm-11-05724-s001.zip › jcm-1884826-supplementary.pdf]

- ONLINE SUPPLEMENTARY FILE -

Respiratory subsets in patients with moderate-to-severe acute respiratory distress syndrome for early prediction of death

Jesús Villar, Cristina Fernández, **Jesús M. González-Martín**, **Carlos Ferrando**, **José M. Añón**, **Ana M. del Saz-Ortíz**, **Ana Díaz-Lamas**, **Ana Bueno-González**, **Lorena Fernández**, **Ana M. Domínguez-Berrot**, **Eduardo Peinado**, **David Andaluz-Ojeda**, **Elena González-Higueras**, **Anxela Vidal**, **M. Mar Fernández**, **Juan M. Mora-Ordoñez**, **Isabel Murcia**, **Concepción Tarancón**, **Eleuterio Merayo**, **Alba Pérez**, **Miguel A. Romera**, **Francisco Alba**, **David Pestaña**, **Pedro Rodríguez-Suárez**, **Rosa L. Fernández**, **Ewout W. Steyerberg**, **Lorenzo Berra**, **Arthur S. Slutsky**

For the Spanish Initiative for Epidemiology, Stratification and Therapies of ARDS (SIESTA) network

Principal Investigator and corresponding author:
Jesús Villar
Multidisciplinary Organ Dysfunction Evaluation Research Network
Hospital Universitario Dr. Negrín
Barranco de la Ballena s/n - 4th floor, South wing
35019 Las Palmas de Gran Canaria. Spain.
E-mail: jesus.villar54@gmail.com

This Supplementary File was provided by the authors to give readers additional information about their work.

RESPIRATORY SUBSETS – Supplementary Material

SIESTA Network Investigators page 3

1. List of centers and investigators involved in the study page 3
2. Author’s contributions page 7

SUPPLEMENTARY METHODS page 8

Ethical aspects page 8

Justification of the study page 8

Patient population page 9

Study design page 10

General care page 11

Data collection and follow-up page 12

Statistical analysis plan page 13

- Sample size page 13
- Predefined rules page 13
- Outcomes page 14
- External validation page 14
- Data analysis page 15

Study organization page 15

- Design and organization page 15
- Funding/support page 16
- Declaration of interests page 17
- Coordination, conduct of the study, and study monitoring page 17

SUPPLEMENTARY RESULTS page 18

Tables S1 to S7 page 20 to page 26

Figures S1 and S2 page 27 to page 28

SUPPLEMENTARY DISCUSSION page 29

SUPPLEMENTARY REFERENCES page 31

SIESTA Network Investigators

1. List of centers and investigators involved in the study periods

Centers	Department/Hospital	Address and city/province	Investigators
1	Intensive Care Unit,<br>Hospital Universitario de La Paz	Paseo de la Castellana 261<br>28046 Madrid, Spain	José M. Añón<br>Belén Civantos<br>Mónica Hernández
2	Post-Surgical Care Unit,<br>Hospital Clínico Universitario de Valencia	Blasco Ibáñez 17<br>46010 Valencia, Spain	Carlos Ferrando*<br>Blanca Arocas<br>Javier Belda<br>Marina Soro<br>Gerardo Aguilar<br>Ernesto Pastor<br>Andrea Gutiérrez
3	Intensive Care Unit,<br>Hospital Universitario Virgen de Arrixaca	Ctra. Madrid-Cartagena s/n<br>30120 El Palmar, Murcia, Spain	Domingo Martínez<br>Juan A. Soler<br>Ana M. del Saz-Ortiz<br>Luís A. Conesa-Cayuela
4	Intensive Care Unit,<br>Hospital Universitario de A Coruña	As Xubias 84<br>15006 A Coruña, Spain	Fernando Mosteiro<br>Lidia Pita-García<br>Ana M. Díaz-Lamas<br>Regina Arrojo
5	Intensive Care Unit,<br>Hospital General Universitario de Ciudad Real	Obispo Rafael Torija s/n<br>13005 Ciudad Real, Spain	Alfonso Ambrós<br>Rafael del Campo<br>Ana Bueno-González<br>Carmen Hornos-López<br>Carmen Martín-Rodríguez
6	Intensive Care Unit,<br>Hospital Universitario Río Hortega	Dulzaina 2<br>47012 Valladolid, Spain	Lorena Fernández<br>Jesús Sánchez-Ballesteros<br>Jesús Blanco<br>Arturo Muriel<br>Pablo Blanco-Schweizer<br>José Ángel de Ayala
7	Intensive Care Unit,<br>Complejo Asistencial Universitario de León	Altos de Nava s/n<br>24001 León, Spain	F. Javier Díaz-Domínguez<br>Demetrio Carriedo<br>Raul I. González Luengo<br>Ana M. Domínguez-Berrot

8	Intensive Care Unit,<br>Hospital Virgen de La Luz	Hermandad Donantes de<br>Sangre 1 16002 Cuenca, Spain	Elena González<br>Rosario Solano<br>María J. Bruscas
9	Intensive Care Unit,<br>Hospital Universitario NS de Candelaria	Ctra. Del Rosario 145<br>38010 Santa Cruz de Tenerife,<br>Spain	Raquel Montiel<br>Dácil Parrilla<br>Eduardo Peinado<br>Lina Pérez-Méndez
10	Intensive Care Unit,<br>Hospital Clínico Universitario de<br>Valladolid	Avda. Ramón y Cajal 3<br>47003 Valladolid, Spain	Leonor Nogales<br>David Andaluz-Ojeda<br>Laura Parra
11	Intensive Care Unit,<br>Hospital Fundación Jiménez Díaz	Avda. Reyes Católicos 2<br>28040 Madrid, Spain	Anxela Vidal<br>Denis Robaglia<br>César Pérez
12	Intensive Care Unit,<br>Hospital Universitario Regional	*Carlos Haya* s/n<br>29010 *Málaga, Spain*	Juan M. Mora-Ordoñez<br>J. Francisco Martínez-<br>Carmona<br>Álvaro Valverde-Montoro<br>Victoria Olea-Jiménez
13	Intensive Care Unit,<br>Hospital Universitario Mutua Terrassa	Plaça del Dr. Robert 5<br>08221 Terrassa, Barcelona,<br>Spain	M. del Mar Fernández
14	Intensive Care Unit,<br>Hospital Universitario de Albacete	Hermanos Falcó 37<br>02006 Albacete, Spain	Isabel Murcia<br>Ángel E. Pereyra-Pache<br>José M. Gutiérrez (retired)<br>Virgilio Córcoles (retired)
15	Intensive Care Unit,<br>Hospital Virgen de la Concha	Avda. Requejo 35<br>49022 Zamora, Spain	Concepción Tarancón<br>Silvia Cortés-Díaz
16	Intensive Care Unit,<br>Hospital Universitario 12 de Octubre	Avda. de Córdoba s/n<br>28041 Madrid, Spain	Isidro Prieto<br>Mario Chico<br>Darío Toral
17	Intensive Care Unit,<br>Hospital NS del Prado	Ctra Madrid Km 114, 45600<br>Talavera de la Reina, Toledo,<br>Spain	Francisco Alba<br>Ruth Corpas
18	Intensive Care Unit,<br>Hospital Universitario Puerta de Hierro	Manuel de Falla 1<br>28222 Majadahonda, Madrid,<br>Spain	Miguel A. Romera<br>Carlos Chamorro-Jambrina
19	Intensive Care Unit,	Médicos sin Fronteras 7	Eleuterio Merayo

	Hospital El Bierzo, Ponferrada	24404 Ponferrada, León, Spain	Chanel Martínez-Jiménez<br>Ángeles de Célis-Álvarez
20	Post-operative Care Unit,<br>Hospital Universitario Río Hortega	Dulzaina 2<br>47012 Valladolid, Spain	César Aldecoa<br>Alba Pérez<br>Jesús Rico-Feijoo<br>Silvia Martín-Alonso
21	Intensive Care Unit,<br>Hospital La Mancha Centro	Avda. Constitución 3,<br>13600 Alcázar de S. Juan,<br>Ciudad Real, Spain	Carmen Martín-Delgado<br>Antonio García (retired)
22	Post-operative Care Unit,<br>Hospital Universitario Ramón y Cajal	Ctra. Colmenar Viejo Km 9.1<br>28034 Madrid, Spain	David Pestaña<br>Adrián Mira<br>Pilar Cobeta
23	Intensive Care Unit,<br>Hospital Univ. Morales Meseguer	Marqués de los Vélez s/n<br>3008 Murcia	Lucia Capilla **
24	Intensive Care Unit,<br>Hospital Público Santa Bárbara	Malagón 0, 13500 Puertollano,<br>Ciudad Real	Francisca Prieto<br>Antonio M. García-Fernández
25	Intensive Care Unit,<br>Hospital General de Segovia	Miguel Servet s/n<br>40002 Segovia	Alec Tallet<br>Santiago Macias<br>Noelia Lázaro
26	Intensive Care Unit,<br>Corporació Sanitaria Parc Tauli	Parc Taulí 1<br>08208 Sabadell, Barcelona	Lluis Blanch<br>Gemma Gomá<br>Gisela Pili
27	Intensive Care Unit,<br>Complejo Hospitalario Universitario de Santiago (Hospital de Conxo)	Ramón Baltar s/n<br>15706 Santiago de Compostela, La Coruña	Antonio Santos-Bouza<br>Cristina Dominguez
28	Intensive Care Unit,<br>Hospital General Río Carrión	Avda. Donantes de Sangre s/n<br>34005 Palencia	Javier Collado<br>José I. Alonso
29	Intensive Care Unit,<br>Hospital General Yagüe	Avda. del Cid campeador, 96<br>09005 Burgos	Alberto Indarte<br>María E. Perea
30	Intensive Care Unit,<br>Hospital de Hellín	Juan Ramón Jiménez, 40<br>02400 Hellín, Albacete	Ricardo Fernández<br>José I. Lozano
31	Intensive Care Unit,<br>Hospital Clínico Universitario de Salamanca	Paseo de San Vicente 182<br>37007 Salamanca	Noelia Albalá<br>Ángel Rodríguez-Encinas
32	Intensive Care Unit<br>Hospital General de Soria	Paseo de Santa Bárbara s/n<br>42005 Soria	Raúl Sánchez<br>Fabiola Tena

33	Research Unit,<br>Hospital Universitario Dr. Negrín	Barranco de la Ballena s/n.<br>35019 Las Palmas de Gran Canaria, Spain	Jesús Villar<br>Jesús M. González-Martín<br>Rosa L. Fernández<br>Cristina Fernández<br>Pedro Rodríguez-Suárez<br>(not enrolling patients)
34	Department of Biomedical Data Sciences,<br>Leiden University Medical Center	P.O. Box 9600, 2300 RC<br>Leiden, The Netherlands	Ewout W. Steyerberg<br>(not enrolling patients)
35	Department of Respiratory Care,<br>Massachusetts General Hospital	55 Fruit St., Warren 1225<br>Boston, Massachusetts 01460. USA	Robert M. Kacmarek (deceased)<br>Lorenzo Berra<br>(not enrolling patients)
36	Li Ka Shing Knowledge Institute, St. Michael’s Hospital	209 Victoria St, Toronto, Ontario M5B 1T8, Canada	Arthur S. Slutsky<br>(not enrolling patients)

(*) current affiliation: Department of Anesthesia and Critical Care, Hospital Clinic, Barcelona, Spain.

(**) current affiliation: Intensive Care Unit, Hospital General Universitario Rafael Méndez, Lorca, Murcia

2. Author's contributions

J. Villar, C. Fernández, R.L. Fernández, and J.M. González-Martín had full access to all study data and takes responsibility for the integrity of the data and accuracy of the data analysis.

Study concept and design: J. Villar, R.M. Kacmarek (deceased prior to preparation of the manuscript), L. Berra, E.W. Steyerberg, C. Ferrando, A.S. Slutsky.

Obtained funding for the study: J. Villar, J.M. Añón, C. Ferrando, P. Rodríguez-Suárez

Acquisition, analysis or interpretation of data: All authors contributed to the final study design, or participated in its coordination, or participated in drafting the first manuscript. C. Ferrando, J.M. Añón, A.M. del Saz-Ortíz, A. Díaz-Lamas, A. Bueno-González, L. Fernández, A.M. Domínguez-Berrot, E. Peinado, D. Andaluz-Ojeda, E. González-Higueras, A. Vidal, M.M. Fernández, J.M. Mora-Ordoñez, I. Murcia, C. Tarancón, E. Merayo, A. Pérez, M.A. Romera, F. Alba, and D. Pestaña enrolled patients into the study and participated in the data collection, data analysis, and the final draft of the manuscript. J. Villar, J.M. González-Martín, C. Fernández, R.L. Fernández, E.W. Steyerberg, L. Berra, and A.S. Slutsky are responsible for data analysis and interpretation of data.

First drafting of the manuscript: J. Villar, C. Fernández, J.M. González-Martín, E.W. Steyerberg, Lorenzo Berra, A.S. Slutsky.

Critical revision for intellectual content and final approval of manuscript: J. Villar, C. Fernández, J.M. González-Martín, C. Ferrando, J.M. Añón, A.M. del Saz-Ortíz, A. Díaz-Lamas, A. Bueno-González, L. Fernández, A.M. Domínguez-Berrot, E. Peinado, D. Andaluz-Ojeda, E. González-Higueras, A. Vidal, M.M. Fernández, J.M. Mora-Ordoñez, I. Murcia, C. Tarancón, E. Merayo, A. Pérez, M.A. Romera, F. Alba, D. Pestaña, P. Rodríguez-Suárez, R.L. Fernández, E.W. Steyerberg, Lorenzo Berra, and A.S. Slutsky. All authors read and approved the final manuscript.

Statistical analysis: J.M. González-Martín, E.W. Steyerberg, J. Villar, A.S. Slutsky, R.L. Fernández, C. Fernández.

Administrative, technical or material support: J. Villar, P. Rodríguez-Suárez, C. Fernández, R.L. Fernández.

Study supervision: J. Villar, C. Fernández, C. Ferrando, J.M. Añón, L. Berra, A.S, Slutsky.

SUPPLEMENTARY METHODS

Ethical aspects

This ancillary study was conducted according to the principles of the Declaration of Helsinki approved by the World Medical Association [1], the European Convention for the Protection of Human Rights and Dignity of the Human Being with regard to the application of Biology and Medicine, and within the requirements established by Spanish legislation for biomedical research, the protection of personal data, and bioethics. Our studies were approved by the Ethics Committees for Clinical Research at the Hospital Universitario Dr. Negrín (Las Palmas de Gran Canaria, Spain, approval No. 2008-0915-EPI), Hospital Virgen de La Luz (Cuenca, Spain, approval No. 2014/PI 1114), Hospital Clínico Universitario de Valladolid (Valladolid, Spain, approval No. PI17-594), Hospital Universitario La Paz (Madrid, Spain, #PI-2694), and adopted by all participating centers, as required by Spanish legislation. The studies were granted a waiver of the need for informed consent, although two sites (Hospital Universitario Ramón y Cajal, Madrid, Spain; Hospital Virgen de la Concha, Zamora, Spain) required informed consent as per the institution's policies. Patient data were anonymized and recorded in a secure, computer-based case report form specifically designed for the study. None of the findings reported in the present study have been published elsewhere. The study followed the STROBE (Strengthening the Reporting of Observational Studies in Epidemiology) guidelines for observational cohort studies [2].

Justification of the study

This ancillary study is an extension of the Spanish Initiative for Epidemiology, Stratification and Therapies of ARDS (SIESTA) Program [3-5].

In 1977, Forrester et al [6] classified 200 patients with acute myocardial infarction (AMI) into four hemodynamic subsets based on a threshold value for cardiac index (2.2 liters/min per m^2) and pulmonary capillary pressure (18 mmHg). Although patients had a wide degree variability in the left ventricular function, the level of cardiac performance assessed by only these two hemodynamic variables had a direct relation to mortality. These four subsets of AMI were of substantial value because in addition to assessing short-term prognosis, each subset established a distinct level of optimal care. The management of patients with AMI has changed dramatically and mortality has decreased over the years, but this classification is still in use because it predicts mortality for each subset of AMI, independently of the patient's age, gender, precipitating factors, and location of the myocardial infarction. Therapeutic interventions resulted in a substantial increase of cardiac index and decrease of

pulmonary capillary pressures, resulting in resolution of clinical abnormalities that paralleled the hemodynamic improvement in more than two-thirds of AMI patients.

To this end, we investigated whether a threshold value of 150 mmHg for PaO_2/FiO_2 and of 10 cmH_2O for positive end-expiratory pressure (PEEP) would classify a large population of mechanically ventilated patients with moderate-to-severe acute respiratory distress syndrome (ARDS) into subsets for predicting mortality in the intensive care unit (ICU). Each subset could represent a category, subphenotype or subclass within the broader phenotype of ARDS population. Each identified subset assessed at 24 hours after moderate/severe ARDS diagnosis was characterized by a pattern of measurable properties (response of PaO_2 under a standardized level of PEEP and FiO_2) that markedly differs from the current ARDS definition [7].

Patient population

This ancillary analysis was conducted in two steps. For the first step, we performed a secondary analysis in an unrestricted set of pooled data from 1,000 adult patients, included prospectively in three multicenter, observational cohorts, enrolling consecutive patients with moderate-to-severe ARDS [7]. Patients were managed with lung-protective mechanical ventilation (MV) and admitted into a network of ICUs in Spain under the SIESTA Program [3-5,8-10]. In the ALIEN cohort [3] (ClinicalTrials.gov NCT00736892. Registered 18 August 2008), 22 participating ICUs included 300 patients from September 2008 to May 2010, from which 255 patients were used to estimate the 1-year incidence of moderate/severe ARDS in 13 geographical areas of Spain. In the STANDARDS cohort (ClinTrials.gov NCT02288949. Registered 13 November 2014), 24 participating ICUs included 300 patients from September 2013 to July 2015, and were used to quantify the risk of death in ARDS [8] and for testing whether driving pressure was superior to the variables that define it in predicting outcome in ARDS patients [9]. The STANDARDS-2 cohort was designed as a continuation of the STANDARDS cohort with the purpose of having a large database of ARDS patients combining our collective efforts. In the STANDARDS-2 cohort (NCT02836444. Registered 19 July 2016), 21 participating ICUs included 400 patients from August 2015 to April 2017, and were used to determine whether an enrichment strategy could be useful for selecting patients into future clinical trials [10].

For the second step, we confirmed the classification model in fully new patients. We analyzed a cohort of 303 consecutive patients with moderate-to-severe ARDS included in the multicenter, observational “Prevalence AND Outcome of acute hypoxemic Respiratory fAilure (PANDORA)” study

(ClinTrials.gov NCT03145974. Registered 9 May 2017). These patients were admitted in a network of 22 ICUs from May 2017 to March 2018 (distributed in 3 periods of two consecutive months) [11]. With this approach, we studied the temporal aspect of external validity since this new cohort contains recently treated ARDS patients (last patient was discharged from hospital on November 1st, 2018). From those patients, 301 patients were used as an external validation cohort for developing an ARDS score [12].

Study design

Patients admitted to participating ICUs were screened daily during the study periods. All consecutive patients meeting the American-European Consensus Conference (AECC) criteria for ARDS [13] on positive end-expiratory pressure (PEEP) ≥5 cmH_2O (in the ALIEN cohort) and the Berlin criteria for moderate or severe ARDS [7] (in the STANDARDS, STANDARDS-2, and PANDORA cohorts) were included. By leaving the assessment of PaO_2/FiO_2 essentially unchanged, the AECC definition and the Berlin criteria are essentially identical [7]. The requirement of a minimum PEEP level of 5 cmH_2O has no impact on the definition since most patients with ARDS are managed with PEEP>5 cmH_2O. Thus, our screening applies only to patients with moderate-to-severe ARDS, which include: (i) having an initiating clinical condition (pneumonia, aspiration, inhalation injury, overdose, sepsis, trauma, acute pancreatitis, etc.), (ii) within one week of a known clinical insult or new or worsening respiratory symptoms, (iii) bilateral pulmonary infiltrates on chest imaging (chest radiograph or computed tomography scan), (iv) absence of left atrial hypertension or no clinical signs of left heart failure, and (v) hypoxemia (as defined by a PaO_2/FiO_2 ≤100 mmHg on PEEP≥5 cmH_2O for severe ARDS, and 100< PaO_2/FiO_2 ≤200 mmHg on PEEP≥5 cmH_2O for moderate ARDS, regardless of FiO_2). We did not enroll patients with persistent mild ARDS (PaO_2FiO_2>200 mmHg during the entire ICU stay). However, we are confident that no patients with mild ARDS were excluded during our observational periods if they moved to a more severe category, although we do not have data on the precise number of those patients.

Day 0 (onset or diagnosis of moderate/severe ARDS) was defined as the day in which the patient first met moderate/severe ARDS criteria, irrespective of day of ICU admission or initiation of MV. All patients had arterial blood gases at study inclusion. We did not use SpO_2 as a surrogate for PaO_2 for enrolling patients. For the purpose of this study and for appropriate identification of patients with ARDS, attending physicians considered only qualifying blood gases while patients were clinically stable, and did not consider transient falls in PaO_2 resulting from acute events unrelated to the disease process (such as obstruction of endotracheal tube by secretions, endotracheal suctioning, ventilator

disconnection, or sudden pneumothorax, among others). We excluded patients younger than 18 years old, patients with severe chronic pulmonary disease, acute cardiac failure, brain death, patients with a do-not-resuscitate orders, or postoperative patients receiving MV for <24 hours. Also, because diagnostic confusion could occur with other diseases and clinical situations that cause hypoxemia and have bilateral pulmonary infiltrates on radiographs, physicians excluded lymphangitic carcinoma, acute eosinophilic pneumonia, hypersensitivity pneumonitis, idiopathic pulmonary fibrosis, and others [14].

General care

Although patient care was not strictly protocolized, clinicians followed current guidelines for the general critical care management, which included the following: (i) in case of sepsis, physicians were urged to ensure early identification of causative microorganism, intravenous administration of antibiotics as soon as sepsis was suspected or recognized, and to optimize antibiotic selection and timely administration on the basis of antibiogram; (ii) fluid resuscitation and vasopressor use were individualized with the goal of maintaining a systolic blood pressure ≥90 mmHg or a mean arterial pressure ≥65 mmHg; (iii) to maintain hemoglobin between 7 to 10 g/dL.

For ventilatory management, clinicians followed current recommendations for lung-protective MV with a tidal volume (VT) of 4-8 ml/kg predicted body weight (PBW), a plateau pressure (Pplat) <30 cmH_2O, a ventilatory rate (RR) to maintain a $PaCO_2$ between 35-50 mmHg (permissive hypercapnia was allowed to target VT), and PEEP and FiO_2 combinations according to the PEEP-FiO_2 table of the ARDS protocol [15], ensuring that among the PEEP and FiO_2 combinations, clinicians should use the PEEP levels that allowed the reduction of FiO_2 to the lowest level for maintaining a PaO_2 within a target range of 60 to 100 mmHg or a peripheral capillary oxygen saturation (SpO_2) within a target range of 90 to 98%.

The choice of drugs for sedation and analgesia, early neuromuscular blockade, prone positioning, recruitment maneuvers, hemodynamic management modalities, and the decision to perform a tracheotomy were left to the discretion of the attending physician. PBW was calculated using the following equations: 50 + 0.91 x [height (cm) – 152) for men and 45.5 + 0.91 x [height (cm) – 152] for women [15]. None of the patients were included in any clinical trial. Although prone positioning and recruitment maneuvers were used in some patients, we do not have data on timing of prone positioning, or whether prone ventilation and recruitment maneuvers were applied as a rescue therapy, as a routine practice, or following any specific protocol.

Weaning off MV was not strictly protocolized, but could be started when the attending physician considered it clinically appropriate. Patients were assessed daily for readiness for a spontaneous breathing trial (SBT) based on the ARDSnet protocol [15]. In general, pre-requisites for the SBT included a partial reversal of the underlying cause of ARDS, a PaO_2/FiO_2>200 with PEEP<10 cmH_2O and FiO_2≤0.4, no vasopressors, continuous sedation minimized, and ability to cough during tracheal aspirations. Spontaneous ventilation was tested with a T-piece or with pressure support at 8 cmH_2O. The duration of the SBT was at least 30 min and no longer than 120 minutes. If the patient passed the trial, a decision for extubation was taken, unless there was a specific reason not to extubate. Weaning and the decision to extubate were left to the discretion of the responsible clinician. Decisions about noninvasive ventilation, reintubation, or extubation were dictated by common clinical criteria.

Data collection and follow-up

Data were collected in each participating ICU using standardized case report forms, and transmitted to the coordinating center (Hospital Universitario Dr. Negrin) when the patient was discharged from hospital. Before exporting the data into a computerized database, a trained data collector from the coordinating center checked the completeness and the quality of information. Logical checks were performed for missing data and to find inconsistencies, especially regarding clinical diagnosis, date, and severity scores. If necessary, the data collector contacted the investigator by phone to validate the data or reformat the data for entry into the database.

For the purpose of this study, we analyzed information from clinical variables including demographics, main cause of ARDS, Acute Physiology And Chronic Health Evaluation II (APACHE II) score during the first 24 hours of ARDS diagnosis (range 0 to 71, with higher scores indicating an increased risk of death) [16], gas exchange (PaO_2, $PaCO_2$, FiO_2, PaO_2/FiO_2, pH), at ARDS diagnosis/onset and at 24 hours of ARDS diagnosis, and data from ventilator settings and lung mechanics (VT, RR, PEEP, peak inspiratory pressure, Pplat), We calculated driving pressure (Pplat minus PEEP) in all patients. At the time of ARDS diagnosis, all patients were ventilated with PEEP≥5 cmH_2O. For the purpose of this study, at 24 hours after meeting moderate/severe ARDS criteria, PaO_2/FiO_2 and Pplat were assessed in all patients (with the exception of those who died before 24 hours of enrollment) under a standardized ventilatory setting (PEEP=10 cmH_2O and FiO_2=0.5) which is standard of practice in these hospitals [17]. When patients required PEEP>10 or FiO_2>0.5 and could not tolerate a decrease in PEEP or FiO_2, a set of rules for setting PEEP and FiO_2 were applied *only*

during the standardized assessment, as described and validated previously by our group [10,17]. At other times, PEEP and FiO_2 levels were up to the discretion of managing clinicians. We recorded organ dysfunction using the sequential organ failure assessment (SOFA) score (range 0 to 4 in each of six domains, with higher scores indicating increasing organ dysfunction) [18] at ARDS onset and after 24 hours of treatment. The six organ systems included respiratory, cardiovascular, liver, kidney, coagulation, and central nervous system. The baseline SOFA score was assumed to be zero in patients not known to have preexisting organ dysfunction. Sepsis was defined according to 2001 International Consensus Conference criteria [19] for the testing cohort of 1000 patients, and according to the Third International Consensus Definition [20] for the confirmatory cohort of 303 patients. In the context of moderate/severe ARDS, all patients had a SOFA score ≥3 at study entry [21].

We recorded the actual duration of MV, the length of stay in the ICU and the length of stay in the hospital. Ventilator-free days (VFDs) were defined as the number of days alive and free from MV from day of inclusion into the study (diagnosis of moderate or severe ARDS) to 28-day. We used the following considerations in calculating VFDs: (i) successful liberation from MV should last >48 hours without reintubation in a 28-day survivor; (ii) extubations only count from the last successful extubation for a 28-day survivor; (iii) VFDs were awarded zero days if the patient was ventilated for ≥28 days or died before 28 days (irrespective of intubation status) [22]. Patients were followed-up until ICU and hospital discharge. We recorded date and status (alive or dead) of the patient at ICU and hospital discharge, and causes of ICU death (refractory hypoxemia, septic shock, multisystem organ failure, brain death, limitation of therapeutic efforts for end-of-life, and others).

Statistical analysis plan

Sample size

Since this was a secondary analysis of four observational studies with no harm and no benefit, we had no predefined sample size requirements for the testing cohort. The confirmatory cohort with fully new patients had a sufficient number of events (112 ICU deaths) required for external validation [23].

Predefined rules and pre-specified statistical analysis

We defined and specified in advance rules and expectations of statistical interpretation before the final statistical analysis was conducted [24]. We realized that overly detailed analysis could produce overoptimistic results due to a combination of reduced statistical power to detect real differences, or due

to an increase in the variance around the mean estimate, and/or an increased statistical likelihood of a false finding when many subgroups are examined.

First, for selecting patients and thresholds for risk of ICU death, we separated patients into subsets based on thresholds values for PaO_2/FiO_2 (150 mmHg) and applied PEEP (10 cmH_2O) at the time of ARDS diagnosis/onset and at 24 hours. We classified patients in the testing and confirmatory cohorts into four possible groups or subsets: Subset I, patients with $PaO_2/FiO_2 \geq 150$ mmHg on PEEP<10 cmH_2O; Subset II, patients with $PaO_2/FiO_2 \geq 150$ on PEEP≥10; Subset III, patients with $PaO_2/FiO_2 < 150$ on PEEP<10; Subset IV, patients with $PaO_2/FiO_2 < 150$ on PEEP≥10. We recorded the values of PaO_2/FiO_2 and PEEP based on the individualized target for PaO_2, PEEP, and FiO_2 levels selected by the attending physician for each individual patient, following the recommendations for ventilatory support of ARDS patients. We did not exclude any patient ventilated with PEEP<10 cmH_2O at 24 hours due to the absence of the site investigator or because the clinician determine that it was in the best interest of the patient not to apply these settings.

Second, we tried to stratify the combined ranges of PaO_2/FiO_2 and PEEP into few prognostic subsets with clear separation of survival from the first 24 h of diagnosis of moderate-to-severe ARDS.

Third, we expected to find ICU mortality differences among subsets at a significance level of <0.005, as recently recommended [25].

Fourth, when all patients were aggregated (n=1303), we expected to find at least 100 patients or 50 deaths in most subsets assessed at 24 h. The decision for a minimum number of 50 deaths was based on a review of 159 randomized clinical trials that tested a variety of intervention on patients with ARDS [26].

Outcomes

Primary outcome was all-cause ICU mortality. Secondary outcomes included number of VFDs to day-28 after moderate/severe ARDS diagnosis and 30-day cumulative survival in the ICU, among others.

External validation

For solving the complexity of confirming the clinical classification model tested in 1000 patients enrolled in three independent cohorts, we conducted a confirmatory validation of the classification model in fully new patients. We analyzed a cohort of 303 patients with moderate-to-severe ARDS included in a multicenter, observational “Prevalence AND Outcome of acute hypoxemic Respiratory fAilure

(PANDORA) study [11]. With this approach, we studied the temporal aspect of external validity since the new external confirmatory cohort contains a population of treated patients with moderate-to-severe ARDS admitted in a network of 22 ICUs during a different time-period (from May 2017 to March 2018). The confirmatory cohort had a sufficient number of events (112 ICU deaths) required for validation [23].

Data analysis

Quantitative variables are expressed as means ± standard deviation (SD), and median and 25-75% percentiles ($P_{25\text{-}75}$). We used the Kolmogorov-Smirnov test to check for normal distribution of data. We used the Student's t test or Mann-Whitney test to compare two numerical variables, and ANOVA test to compare more than two numerical variables. We used Fisher's exact test or Pearson Chi-squared test for analyzing differences between categorical variables. We examined the probability of ICU survival to day-30 for the initial four subsets identified in the entire population of 1303 moderate/severe ARDS patients, and for the two global subsets of patients with PaO_2/FiO_2 <150 mmHg and PaO_2/FiO_2 ≥150 mmHg at 24 hours of ARDS diagnosis using the Kaplan-Meier method with the log-rank test. Patients discharged alive from ICU before day-30 of study inclusion were censored. No assumptions were made for missing data. We also calculated the differences between means, risk ratio (RR), hazard ratio (HR), odds ratio (OR), and the 95% confidence intervals (CI). The 95%CIs for the difference between medians were estimated using a bootstrap procedure (10,000 replications).

We used dot plots to present distributions of patients based on PaO_2/FiO_2 versus PEEP. By protocol (study design), we chose cutoffs of 150 mmHg for PaO_2/FiO_2 and 10 cmH_2O for PEEP to form the matrices. We performed logistic regression analyses to test the comparison between the threshold of PaO_2/FiO_2<150 mmHg with patient's age and SOFA score at 24 hours in relation to ICU mortality. With the probabilities obtained, we estimated the OR and the area under the receiver operating characteristics curve (AUC). For all comparisons, a two-sided p-value <0.005 was considered to keep a false discovery rate below 5%, as recommended [25]. Analyses were performed using R Core Team 2022 software, version 4.2 (R Foundation for Statistical Computing, Vienna, Austria).

Study organization

Design and organization

Initially, the main investigators of this study were Jesús Villar (principal investigator), Robert M. Kacmarek and Lorenzo Berra (from the Department of Anesthesia, Massachusetts General Hospital,

Boston, USA), José M. Añón (ICU, Hospital Universitario La Paz, Madrid, Spain), and Carlos Ferrando (Hospital Clinic, Barcelona, Spain) who all were involved in the study design, ethical approval, registration at ClinicalTrial.Gov web page, research-in-progress meetings, and the statistical analysis plan. Due to the COVID-19 pandemic and because Robert M. Kacmarek developed a devastating and fatal disease before the overview of data collection and the final statistical analysis, two new investigators, Ewout W. Steyerberg (Department of Biomedical Data Sciences, Leiden University Medical Center, Leiden, The Netherlands) and Arthur S. Slutsky (Li Ka Shing Knowledge Institute at St. Michael's Hospital, Toronto, Canada) joined the team of main investigators with the approval and acceptance of all investigators. The new investigators were involved in a new overview of the study design, reinterpretation of findings, statistical analysis, and drafting the manuscript.

Funding/Support

This ancillary study was an academic, investigator-initiated, non-industry sponsored, non-interventional, multicenter, observational study. There was no specific funding source for this observational study.

The study coordinator and principal investigator (J. Villar) was funded by grants from the Instituto de Salud Carlos III, Madrid, Spain (PI16/00049, PI19/00141), by the European Reginal Development's Funds, and the Asociación Científica Pulmón y Ventilación Mecánica (Las Palmas de Gran Canaria, Spain). J. Villar, J.M. Añón, and C. Ferrando received grant support from the Institute de Salud Carlos III, Madrid, Spain (CB06/06/1088). P. Rodríguez-Suárez was funded by the Health Service of the Autonomic Government of the Canary Islands, and by the Royal College of Physicians of Las Palmas de Gran Canaria, Spain. Funds were exclusively allocated for covering salary support of project data managers. L. Berra receives salary support from K23HL128882/NHLBI NIH, research funds from the Regional Jenny Endowment Chair at Harvard Medical School, the Sunday Funds at Massachusetts General Hospital, and research grants from iNO Therapeutics LLC. A.S. Slutsky was funded by the Canadian Institutes of Health (grants #137772 and FDN 143285). None of the funding agencies supporting the principal investigators participated in the study design, data collection, analysis and interpretation of data, or in the writing and submission of the study report. All researchers are independent of their funding bodies. The authors would like to thank Mr. Yasser and Lily B of LP Behrain for their support in loving memory of Lily Bendahan. Centers enrolling patients were the legal sponsors of the study in their own hospitals.

Declaration of interests

Authors declare no competing interests directly or indirectly related to this study. None of the clinical investigators received any honorarium for participating in the study.

Coordination, conduct of the study, and study monitoring

Since a key lesson in the diagnostic process is synchronous communication (when all local investigators are present at the same time), real-time synchronous exchange of ideas and information, and interdisciplinary meetings to derive plans are invaluable [27]. Before enrolling the first patient in any of the four cohorts used in this study, the principal investigator, the data manager, and all site investigators from participating ICUs attended at least one formal session in Madrid, Spain, for discussing the study protocols and data collection on standardized case report forms (CRF). All documents required for each cohort, including the study protocol, management guidelines, copies of RFs, model of an informed consent form (in case it was needed) were available for each attending investigator at each participating ICUs, to ensure compliance with the daily patient screening and inclusion, with the study protocol, and with the data collection process.

Face-to-face meetings were held as determined by need. Routine research meetings were conducted in Madrid, Spain, or by email, fax, or teleconferencing. Since there are well documented issues of clinician‘s ability to recognize ARDS [28], we monitored through discussion in regular meetings and by contacting site investigators after each study period. In order to avoid confirmation bias, we opted not to use the raw data in the CRF for ARDS identification. An electronic Newsletter for every cohort regularly informed investigators on study conduct and or any relevant information for the study. In every cohort, the coordinating center (Research Unit, Hospital Universitario Dr. Negrin, Las Palmas de Gran Canaria, Spain) took responsibility for: (i) communicating to participating sites, (ii) monitoring and supervising the progress of the cohort, (iii) informing and advising on all aspects of the study.

SUPPLEMENTARY RESULTS

Although the year of patient's admission in the ICU was not accounted for our analysis, the all-cause ICU mortality across the three periods in the testing population of 1000 patients, and the all-cause ICU mortality of the confirmatory cohort were similar: 123/300 (41%, 95%CI 35.4%-46.6%), 114/300 (38%, 95%CI 32.5%-43.5%), 138/400 (34.5%, 95%CI 29.8%-39.2%), and 112/303 (37.0%, 95%CI 31.5%-42.4%) and not significantly different (p=0.366). Each of the three cohorts pooled for the testing dataset and the confirmatory cohort had more than 100 events (ICU deaths). Participating centers did not report any loss of patients with moderate-to-severe ARDS during the study periods. Five patients (4 in the testing cohort and one in the confirmatory cohort) died before the 24 h assessment on standardized ventilator settings. However, since all these five patients had a $PaO_2/FiO_2 \leq 00$ mmHg on PEEP≥10 and $FiO_2 > 0.5$ at the time of death, they were included in the 24 hours analysis.

Testing cohort

Twenty-eight patients were in subset I (23 ICU survivors and 5 non-survivors) (see **Table 3** in the main text). Median PEEP was 8 cmH_2O (P_{25-75} 6-8). From those 23 survivors, 18 patients had PaO_2/FiO_2 >200 mmHg at 24 hours after diagnosis of moderate/severe ARDS, and three patients were weaned at the time of assessment. Only five patients from subset I died in the ICU (**Table S3**). Those five patients died from pulmonary and extrapulmonary organ failure associated with the underlying disease (cancer, acquired immunodeficiency syndrome, severe acute pancreatitis), or advanced age.

Twenty-five patients were in subset III (15 ICU survivors and 10 non-survivors) (**Table 3**). Median PEEP was 8 cmH_2O (P_{25-75} 7-8). From those 15 survivors, one patient had a sudden pneumothorax and one patient had severe hemodynamic instability at the time of assessment at 24 hours. Ten patients from subset III died in ICU. In those 10 patients, one patient died the day of assessment, one developed a tension pneumothorax at the time of assessment, one had severe bronchial rupture by a severe chest trauma, one died from severe brain damage caused by cerebral haemorrhage, and five patients died from multisystem organ failure associated with the underlying disease (cancer, acute pancreatitis).

Confirmatory cohort

APACHE II score was not reported in 104 out of 303 (34.3%) at the time of assessment at 24 hours after diagnosis of moderate-to-severe ARDS. As a result, mean values of APACHE II scores for

each subset of the confirmatory cohort were not analysed for each subset at 24 hours. Twenty-eight patients were in subset I (24 ICU survivors and 4 non-survivors) (**Table S1**). Median PEEP was 8 cmH_2O ($P_{25\text{-}75}$ 7-8). From the 24 survivors, 12 patients had PaO_2/FiO_2 >200 mmHg at 24 h, one patient was weaned from MV at the time of assessment, and one patient was transferred to ECMO at the time of assessment. Only four patients from subset I died in the ICU (**Table S3**): one had severe acute pancreatitis and died at 48 hours of moderate/severe ARDS onset from irreversible shock, one died from limitation of therapeutic efforts, and two patients had terminal cancer and died from refractory hypoxemia and multisystem organ failure.

Fourteen patients were in subset III (7 ICU survivors and 7 non-survivors) (**Table S3**). Median PEEP was 8 cmH_2O ($P_{25\text{-}75}$ 7-8). From the seven survivors, one patient had a PaO_2/FiO_2 ≥150 mmHg on the same day of assessment, one patient was extubated within the first 48 hours of study inclusion, and three patients had a PaO_2/FiO_2 >150 mmHg at 48 hours of study inclusion. From the seven deaths in the ICU, four died on the same day of assessment from multiple trauma or multisystem organ failure, one died 24 hours later from a terminal cancer, one died from multisystem organ failure associated with leukemia, and one patient with multiple chronic diseases died from multisystem organ failure.

Aggregated patient population

Overall all-cause ICU mortality of the entire patient population was 37% (487/1303). Ninety-five patients (7.3% from the entire 1303 study population) were ventilated with PEEP<10 cmH_2O at 24 hours after moderate/severe ARDS diagnosis: 53 patients from the testing cohort (28 in subset I and 25 in subset III) and 42 from the confirmatory dataset (28 in subset I and 14 in subset III).

Using logistic regression analysis, we found that a threshold of PaO_2/FiO_2<150 at 24 hours was better than patient's age and SOFA score on the day of PaO_2/FiO_2 assessment under a standardized ventilator setting in predicting ICU death: OR 3.1, 95%CI 2.4-4.0) (**Table S5**).

Table S1. Sensibility analysis for cross-validation of pooled studies included in the testing cohort (N=1000) according to subsets of patients with moderate-to-severe acute respiratory distress syndrome (ARDS). Classification was made based on PaO_2/FiO_2 values.

	**Group I** $PaO_2/FiO_2 \geq 150$ on PEEP<10	**Group II** $PaO_2/FiO_2 \geq 150$ on PEEP≥10	**Group III** $PaO_2/FiO_2 < 150$ on PEEP<10	**Group IV** $PaO_2/FiO_2 < 150$ on PEEP≥10	**p-value**
**ALIEN cohort (N=300)**					
At moderate/severe ARDS onset					
No. of subjects	28	32	115	125	
No. events (ICU deaths)	6	9	50	58	
Event rate (95%CI)	21.4 (6.2-36.6)	28.1 (12.6-43.7)	43.5 (34.4-52.5)	46.4 (37.7-55.1)	0.039
Risk ratio (95%CI)	1 (Ref)	1.3 (0.5 to 3.2)	2.0 (1.0 to 4.2)	2.2 (1.0 to 4.5)	0.039
At 24 hours after onset					
No. of subjects	14	97	15	174	
No. events (ICU deaths)	4	24	5	90	
Event rate (95%CI)	28.6 (4.9-52.2)	24.7 (16.2-33.3)	33.3 (9.5-57.2)	51.7 (44.3-59.2)	<0.001
Risk ratio (95%CI)	1 (Ref)	0.9 (0.4 to 2.1)	1.2 (0.4 to 3.5)	1.8 (0.8 to 4.2)	<0.001
**STANDARDS cohort (N=300)**					
At moderate/severe ARDS onset					
No. of subjects	30	48	57	165	
No. events (ICU deaths)	12	15	21	66	
Event rate (95%CI)	40.0 (22.5-57.5)	31.3 (18.1-44.4)	36.8 (24.3-49.4)	40.0 (32.5-47.5)	0.732
Risk ratio (95%CI)	1 (Ref)	0.8 (0.4 to 1.4)	0.9 (0.5 to 1.6)	1 (0.6 to 1.6)	0.541
At 24 hours after onset					
No. of subjects	1	143	5	151	
No. events (ICU deaths)	1	31	3	79	
Event rate (95%CI)	100 (2.5-100)	21.7 (14.9-28.4)	60.0 (17.1-100)	52.3 (44.3-60.3)	<0.001
Risk ratio (95%CI)	4.6 (3.4 to 6.3)	1 (Ref)	2.8 (1.3 to 6.0)	2.4 (1.7 to 3.4)	<0.001
**STANDARDS-2 cohort (N=400)**					
At moderate/severe ARDS onset					
No. of subjects	15	55	68	262	
No. events (ICU deaths)	7	17	26	88	
Event rate (95%CI)	46.7 (21.4-71.9)	30.9 (18.7-43.1)	38.2 (26.7-49.8)	33.6 (27.9-39.3)	0.613
Risk ratio (95%CI)	1 (Ref)	0.7 (0.3 to 1.3)	0.8 (0.4 to 1.5)	0.7 (0.4 to 1.3)	0.621
At 24 hours after onset					
No. of subjects	13	163	5	219	
No. events (ICU deaths)	0	37	2	99	
Event rate (95%CI)	0 (0-24.7)	22.7 (16.3-29.1)	40.0 (5.3-85.3)	45.2 (38.6-51.8)	<0.001
Risk ratio (95%CI)	-	1 (Ref)	1.8 (0.6 to 5.3)	2-0 (1.4 to 2.7)	<0.001

ARDS: acute respiratory distress syndrome; CI: confidence interval; ICU: intensive care unit; PEEP: positive end-expiratory pressure

Table S2. Main characteristics of 303 patients with moderate-to-severe acute respiratory distress syndrome (ARDS). Classification was made at 24 h after diagnosis of ARDS as Subset I, II, III, and IV based on cut-off values of 150 mmHg for PaO_2/FiO_2 and 10 cmH_2O for PEEP*.

Variables	Values				
	Subset I N=28	Subset II N=139	Subset III N=14	Subset IV N=122	p-value
Age, mean±SD	61 ± 12	56 ± 16	62 ± 14	60 ± 14	0.082
Gender, No. (%)					0.928
Men	21 (75.0)	100 (71.9)	10 (71.4)	92 (75.4)	
Women	7 (25.0)	39 (28.1)	4 (28.6)	30 (24.6)	
VT, ml/kg PBW					
mean±SD	6.7 ± 1.2	6.7 ± 1.0	6.8 ± 1.2	6.3 ± 1.1	0.015
mean difference (95%CI)	0 (Ref)	0 (-0.4 to 0.4)	0.1 (-0.7 to 0.9)	-0.4 (-0.9 to 0.1)	0.015
Plateau pressure, cmH_2O					
mean±SD	20.7 ± 5.5	24.0 ± 3.9	24.4 ± 4.6	27.2 ± 4.2	<0.001
mean difference (95%CI)	0 (Ref)	3.3 (1.6 to 5.0)	3.7 (0.2 to 7.2)	6.5 (4.7 to 8.4)	<0.001
PEEP, cmH_2O					
mean±SD	7.8 ± 1.5	11.8 ± 2.2	7.4 ± 1.4	12.9 ± 2.7	<0.001
mean difference (95%CI)	0 (Ref)	4.0 (3.1 to 4.9)	-0.4 (-1.4 to 0.6)	5.1 (4.0 to 6.1)	<0.001
Driving pressure, cmH_2O					
mean±SD	13 ± 5	12 ± 3	16 ± 4	14 ± 4	<0.001
mean difference (95%CI)	0 (Ref)	-1 (-2 to 0)	3 (0 to 6)	1 (-1 to 3)	<0.001
FiO_2					
mean±SD	0.51 ± 0.10	0.54 ± 0.10	0.70 ± 0.19	0.74 ± 0.17	<0.001
mean difference (95%CI)	0 (Ref)	0.03 (-0.01 to 0.07)	0.19 (0.10 to 0.28)	0.23 (0.16 to 0.30)	<0.001
PaO_2/FiO_2, mmHg					
mean±SD	198 ± 42	216 ± 54	111 ± 27	106 ± 26	<0.001
mean difference (95%CI)	0 (Ref)	18 (-3 to 39)	-87 (-112 to -62)	-92 (-104 to -80)	<0.001
SOFA score					
mean±SD ¶	9.5 ± 4.7	8.7 ± 3.4	11.1 ± 3.2	11.0 ± 4.3	<0.001
mean difference (95%CI)	0 (Ref)	-0.8 (-2.3 to 0.7)	1.6 (-1.2 to 4.4)	1.5 (-0.3 to 3.3)	<0.001
Days on MV from ARDS diagnosis					
mean±SD	14.8 ± 13.9	14.6 ± 17.8	5.5 ± 4.4	13.0 ± 16.6	0.250
mean difference (95%CI)	0 (Ref)	-0.2 (-7.2 to 6.8)	-9.3 (-17.0 to -1.6)	-1.8 (-8.5 to 4.9)	0.250
VFDs, d					
mean±SD	12.1 ± 9.4	12.5 ± 9.9	9.2 ± 11.4	4.7 ± 7.4	<0.001
mean difference (95%CI)	0 (Ref)	0.4 (-3.6 to 4.4)	-2.9 (-9.6 to 3.8)	-7.4 (-10.6 to -4.2)	<0.001
ICU deaths					
No. events	4	28	7	73	
Event rate (95%CI)	14.3 (1.3-27.3)	20.1 (13.5-26.8)	50.0 (23.8-76.2)	59.8 (51.1-68.5)	<0.001
Risk ratio (95%CI)	1 (Ref)	1.4 (0.5 to 3.7)	3.5 (1.2 to 10.0)	4.2 (1.7 to 10.5)	<0.001

ARDS: acute respiratory distress syndrome; ICU: intensive care unit; MV: mechanical ventilation; PBW: predicted body weight; PEEP: positive end-expiratory pressure; SD: standard deviation; SOFA: sequential organ failure assessment; VFDs: ventilator-free days from moderate/severe ARDS diagnosis to day-28; VT: tidal volume.

¶ SOFA score was not reported in 5 patients (3 patients from Subset II; 2 patients from subset IV).

(*) Subset I ($PaO_2/FiO_2 \geq 150$ on PEEP<10); Subset II ($PaO_2/FiO_2 \geq 150$ on PEEP≥10); Subset III ($PaO_2/FiO_2 < 150$ on PEEP<10); Subset IV ($PaO_2/FiO_2 < 150$ on PEEP≥10).

Table S3. Causes of death in patients with moderate-to-severe acute respiratory distress syndrome from the testing and confirmatory cohorts. Classification was made at 24 hours after diagnosis of moderate/severe acute respiratory distress syndrome as Subsets I, II, III, and IV based on cut-off values of 150 mmHg for PaO_2/FiO_2 and 10 cmH_2O for PEEP*. ICU: Intensive care unit.

Causes and No. of deaths in ICU	Values				
Testing Cohort (n=1000)	Subset I<br>N=28	Subset II<br>N=403	Subset III<br>N=25	Subset IV<br>N=544	p-value
Total No. of deaths (%)	5 (17.9)	92 (22.8)	10 (40)	268 (49.3)	<0.001
Multiple system organ failure, no. (%)	2 (40)	43 (46.7)	5 (50)	129 (48.1)	0.752
Refractory hypoxemia, no. (%)	2 (40)	13 (14.1)	2 (20)	59 (22.0)	0.236
Irreversible septic shock, no. (%)	1 (20)	17 (18.5)	2 (20)	39 (14.6)	0.338
Brain death, no. (%)	-	5 (5.4)	-	14 (5.2)	0.878
Limitation of therapeutic efforts, no. (%)	-	10 (10.9)	-	20 (7.5)	0.490
Others (arrhythmia, cardiogenic shock, haemorrhagic shock, anaphylactic) shock), no. (%)	-	4 (4.3)	1 (10)	7 (2.6)	0.454
Confirmatory Cohort (n=303)	Subset I<br>N=28	Subset II<br>N=139	Subset III<br>N=14	Subset IV<br>N=122	p-value
Total No. of deaths (%)	4 (14.3)	28 (20.1)	7 (50.0)	73 (59.8)	<0.001
Multiple system organ failure	1 (25.0)	16 (57.1)	4 (57.1)	37 (50.7)	0.692
Refractory hypoxemia	1 (25.0)	2 (7.1)	-	12 (16.4)	0.350
Irreversible septic shock	1 (25.0)	3 (10.7)	1 (14.3)	9 (12.3)	0.664
Brain death	-	-	-	1 (1.4)	1
Limitation of therapeutic efforts	1 (25.0)	6 (21.4)	2 (28.6)	9 (12.3)	0.321
Others (ventricular arrhythmia, cardiogenic shock, haemorrhagic shock, anaphylactic) shock)	-	1 (3.6)	-	5 (6.8)	1

(*) Subset I ($PaO_2/FiO_2 \geq 150$ on PEEP<10); Subset II ($PaO_2/FiO_2 \geq 150$ on PEEP≥10); Subset III ($PaO_2/FiO_2 < 150$ on PEEP<10); Subset IV ($PaO_2/FiO_2 < 150$ on PEEP≥10).

Table S4. Main characteristics of 1,303 patients with moderate-to-severe ARDS. Classification was made at 24 hours of moderate/severe ARDS diagnosis based on PaO_2/FiO_2 values.

	**PaO_2/FiO_2 ≥150** N=598	**PaO_2/FiO_2 <150** N=705	p-value
Gender, No. (%)			0.072
Men	399 (66.7)	504 (71.5)	
Woman	199 (33.3)	201 (28.5)	
Age, years			
mean ± SD	56.6 ± 15.6	57.6 ± 15.8	0.252
APACHE II	n=528	n=638	
mean ± SD	17.5 ± 7.3	20.6 ± 7.2	<0.001
mean difference (95%CI)	0 (Ref)	3.1 (2.3 to 3.9)	<0.001
VT, ml/kg PBW			
mean ± SD	6.8 ± 0.9	6.7 ± 1.0	0.060
mean difference (95%CI)	0 (Ref)	-0.1 (-0.2 to 0.1)	0.060
Plateau pressure, cmH_2O			
mean ± SD	24.7 ± 4.6	27.8 ± 4.3	<0.001
mean difference (95%CI)	0 (Ref)	3.1 (2.6 to 3.6)	<0.001
PEEP, cmH_2O			
mean ± SD	11.9 ± 2.9	12.6 ± 3.0	<0.001
mean difference (95%CI)	0 (Ref)	0.7 (0.4 to 1.0)	<0.001
Driving pressure, cmH_2O			
mean ± SD	12.9 ± 4.2	15.1 ± 4.5	<0.001
mean difference (95%CI)	0 (Ref)	2.2 (1.7 to 2.7)	<0.001
FiO_2			
mean ± SD	0.55 ± 0.11	0.75 ± 0.17	<0.001
mean difference (95%CI)	0 (Ref)	0.20 (0.18 to 0.22)	<0.001
PaO_2/FiO_2, mmHg			
mean ± SD	205 ± 48	107 ± 27	<0.001
mean difference (95%CI)	0 (Ref)	-98 (-102 to -94)	<0.001
SOFA score	n=595	n=703	
mean ± SD	8.3 ± 3.5	10.1 ± 3.9	<0.001
mean difference (95%CI)	0 (Ref)	1.8 (1.4 to 2.2)	<0.001
Days on MV in ICU survivors			
mean ± SD	15.5 ± 15.6	21.6 ± 18.5	<0.001
mean difference (95%CI)	0 (Ref)	6.1 (4.2 to 8.0)	<0.001
Ventilator-free days to day-28, d			
mean ± SD	11.6 ± 9.7	5.2 ± 7.9	<0.001
mean difference (95%CI)	0 (Ref)	-6.4 (-7.4 to -5.4)	<0.001
All-cause ICU mortality			
No. events	129	358	
Event rate (95%CI)	21.6 (18.3-24.9)	50.8 (47.1-54.5)	<0.001
Risk ratio (95%CI)	1 (Ref)	2.4 (2.0 to 2.8)	<0.001
All-cause hospital mortality			
No. events	158	381	
Event rate (95%CI)	26.4 (22.9-30.0)	54.0 (50.4-57.7)	<0.001
Risk ratio (95%CI)	1 (Ref)	2.0 (1.8 to 2.4)	<0.001

APACHE: Acute Physiology and Chronic Health Evaluation; ARDS: acute respiratory distress syndrome; ICU: intensive care unit; MV: mechanical ventilation; PBW: predicted body weight; PEEP: positive end-expiratory pressure; SD: standard deviation; SOFA: sequential organ failure assessment; VT: tidal volume.

Table S5. Logistic regression analysis for adjusted impact of PaO_2/FiO_2 at 24 hours on standardized ventilatory settings in relation to patient's age and SOFA score at the time of PaO_2/FiO_2 assessment in 1303 patients with moderate-to-severe ARDS.

	Multivariate analysis				
**Variable**	**Coefficient b**	**Standard Error**	**Odds Ratio**	**95% CI**	**p-value**
(Intercept)	-5.45	0.37	0.004	0.002 - 0.01	<0.001
PaO_2/FiO_2 <150 mmHg at 24 hours on SVS.	1.13	0.14	3.08	2.36 - 4.04	<0.001
Patient's Age	0.04	0	1.04	1.03 - 1.05	<0.001
SOFA score at 24 hours of study inclusion	0.22	0.02	1.24	1.20 - 1.29	<0.001
AUC (95%CI)	0.79 (0.76 – 0.81)				

AUC: area under the curve of the receiver operating characteristics; CI: confidence interval; SOFA: sequential organ failure assessment; SVS: standardized ventilatory setting.

Table S6. Distribution and mortality in the intensive care unit (ICU) of each subset of patients with moderate-to-severe acute respiratory distress syndrome (ARDS) from the testing (n=1,000) and confirmatory (n=303) cohorts using 100 mmHg as a cut-off value for PaO_2/FiO_2.

Cohort	Timing	**Subset I** PaO_2/FiO_2>100 on PEEP<10	**Subset II** PaO_2/FiO_2>100 on PEEP≥10	**Subset III** PaO_2/FiO_2≤100 on PEEP<10	**Subset IV** PaO_2/FiO_2≤100 on PEEP≥10	p-value
**Testing cohort**	**At ARDS onset**					
	No. of subjects	204	386	109	301	
	No. events (ICU deaths	76	127	46	126	
	Event rate (95%CI)	37.3 (30.6-43.9)	32.9 (28.2-37.6)	42.2(32.9-51.5)	41.9(36.3-47.4)	0.064
	Risk ratio (95%CI)	1 (Ref)	0.9 (0.7-1.1)	1.1 (0.9-1.5)	1.1 (0.9-1.4)	0.074
	**At 24 hours after onset**					
	No. of subjects	43	739	10	208	
	No. events (ICU deaths	7	226	8	134	
	Event rate (95%CI)	16.3 (5.2-27.3)	30.6 (27.3-33.9)	80.0 (55.2-100)	64.4(57.9-70.9)	<0.001
	Risk ratio (95%CI)	1 (Ref)	1.9 (0.9-3.7)	2.9 (2.3-10.4)	4.0 (2.0-7.8)	<0.001
**Confirmatory cohort**	**At ARDS onset**					
	No. of subjects	75	121	35	72	
	No. events (ICU deaths	24	40	19	29	
	Event rate (95%CI)	32.0 (21.4-42.6)	33.1 (24.7-41.4)	54.3(37.8-70.8)	40.3(29.0-51.6)	0.093
	Risk ratio (95%CI)	1 (Ref)	1.0 (0.7-1.6)	1.7 (1.1-2.7)	1.3 (0.8-1.9)	0.114
	**At 24 hours after onset**					
	No. of subjects	37	211	5	50	
	No. events (ICU deaths	7	64	4	37	
	Event rate (95%CI)	18.9 (6.3-31.5)	30.3 (24.1-36.5)	80.0 (44.9-100)	74.0(61.8-86.2)	<0.001
	Risk ratio (95%CI)	1 (Ref)	1.6 (0.8-3.2)	4.2 (1.9-9.4)	3.9 (2.0-7.8)	<0.001

ARDS: acute respiratory distress syndrome; ICU: intensive care unit; PEEP: positive end-expiratory pressure.

Table S7. Distribution and mortality in the intensive care unit (ICU) of each subset of patients with moderate-to-severe acute respiratory distress syndrome (ARDS) from the testing (n=1,000) and confirmatory (n=303) cohorts using 120 mmHg as a cut-off value for PaO_2/FiO_2.

Cohorts	Timing	**Subset I** $PaO_2/FiO_2 \geq 120$ on PEEP<10	**Subset II** $PaO_2/FiO_2 \geq 120$ on PEEP≥10	**Subset III** $PaO_2/FiO_2 < 120$ on PEEP<10	**Subset IV** $PaO_2/FiO_2 < 120$ on PEEP≥10	p-value
**Testing cohort**	**At ARDS onset**					
	No. of subjects	156	268	157	419	
	No. events (ICU deaths)	59	83	63	170	
	Event rate (95%CI)	37.8 (30.2-45.4)	31.0 (25.4-36.5)	40.1 (32.5-47.8)	40.6 (35.9-45.3)	0.091
	Risk ratio (95%CI)	1 (Ref)	0.8 (0.6-1.1)	1.1 (0.8-1.4)	1.1 (0.9-1.4)	0.071
	**At 24 hours after onset**					
	No. of subjects	38	622	15	325	
	No. events (ICU deaths)	7	179	8	181	
	Event rate (95%CI)	18.4 (6.1-30.8)	28.8 (25.2-32.3)	53.3 (28.1-78.6)	55.7 (50.3-61.1)	<0.001
	Risk ratio (95%CI)	1 (Ref)	1.6 (0.8-3.1)	2.9 (1.3-6.6)	3.0 (1.5-5.9)	<0.001
**Confirmatory cohort**	**At ARDS onset**					
	No. of subjects	56	98	54	95	
	No. events (ICU deaths)	16	32	27	37	
	Event rate (95%CI)	28.6 (16.7-40.4)	32.7 (23.4-41.9)	50.0 (36.7-63.3)	39.0 (21.1-48.8)	0.097
	Risk ratio (95%CI)	1 (Ref)	1.1 (0.7-1.9)	1.8 (1.1-2.9)	1.4 (0.8-2.2)	0.088
	**At 24 hours after onset**					
	No. of subjects	34	179	8	82	
	No. events (ICU deaths)	5	52	6	49	
	Event rate (95%CI)	14.7 (2.8-26.6)	29.1 (22.4-35.7)	75.0 (45.0-100)	59.8 (49.1-70.4)	<0.001
	Risk ratio (95%CI)	1 (Ref)	2 (0.9-4.6)	5.1 (2.1-12.6)	4.1 (1.8-9.3)	<0.001

ARDS: acute respiratory distress syndrome; ICU: intensive care unit; PEEP: positive end-expiratory pressure.

SUPPLEMENTARY FIGURES

Figure S1. Distribution of 303 patients (confirmatory cohort) with moderate-to-severe acute respiratory distress syndrome (ARDS) based on cutoff values for PaO_2/FiO_2 ratio (150 mmHg) and positive end-expiratory pressure level (10 cmH_2O) for each individual patient. **A)** At the time of moderate/severe ARDS diagnosis (baseline). **B)** After 24 hours of usual critical care with protective mechanical ventilation. The dotted lines are placed at PaO_2/FiO_2 ratio of 150 mmHg and at PEEP of 10 cm H_2O. Mortality increases as lung function deteriorates (from Subset I to Subset IV) at 24 hours. Subset I: $PaO_2/FiO_2 \geq 150$ on PEEP<10; Subset II: $PaO_2/FiO_2 \geq 150$ on PEEP≥10; Subset III: $PaO_2/FiO_2 < 150$ on PEEP<10; Subset IV: $PaO_2/FiO_2 < 150$ on PEEP≥10.

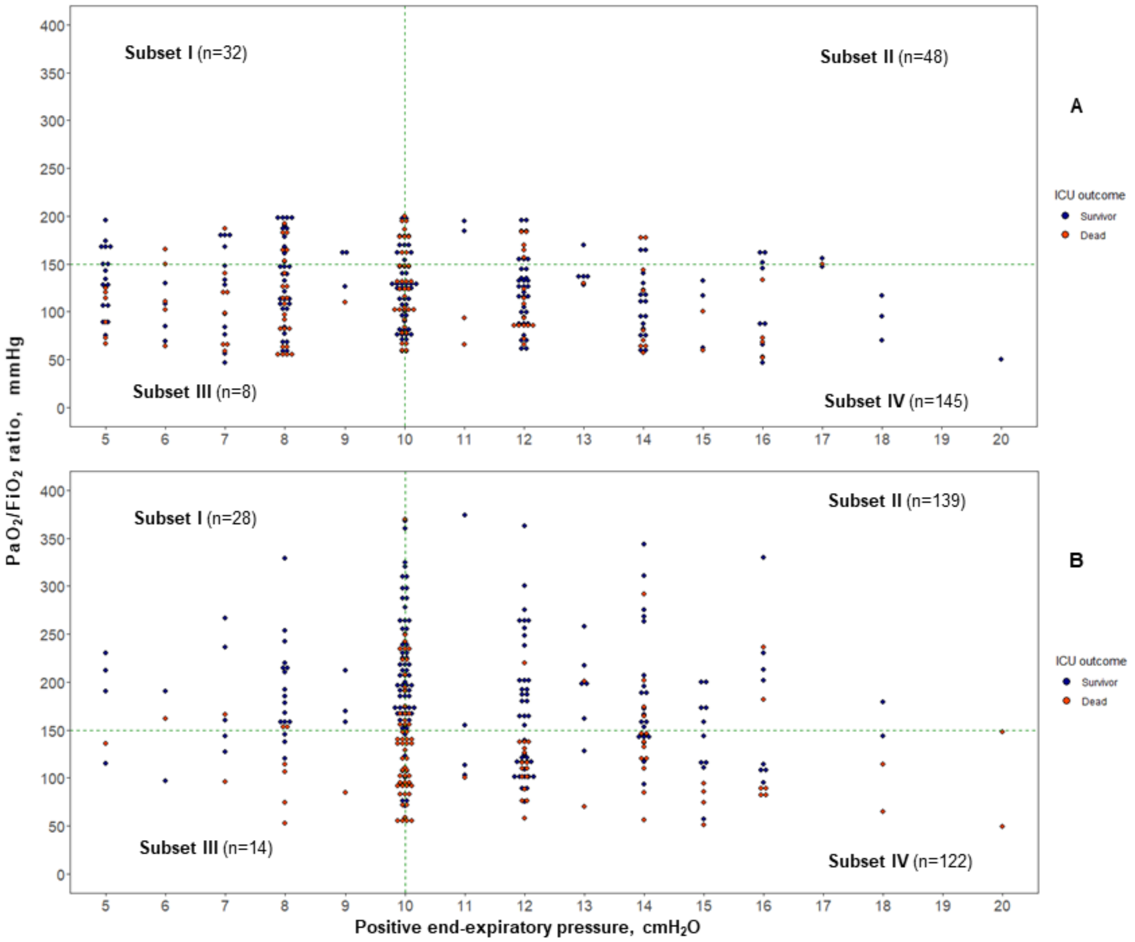

Figure S2. Probability of cumulative ICU survival to day-30 in 1303 patients with moderate-to-severe acute respiratory distress syndrome (ARDS). Patients were stratified into 4 subsets based on cut-off values of 150 mmHg for PaO_2/FiO_2 and 10 cmH_2O for positive end-expiratory pressure at 24 hours of moderate/severe ARDS diagnosis.

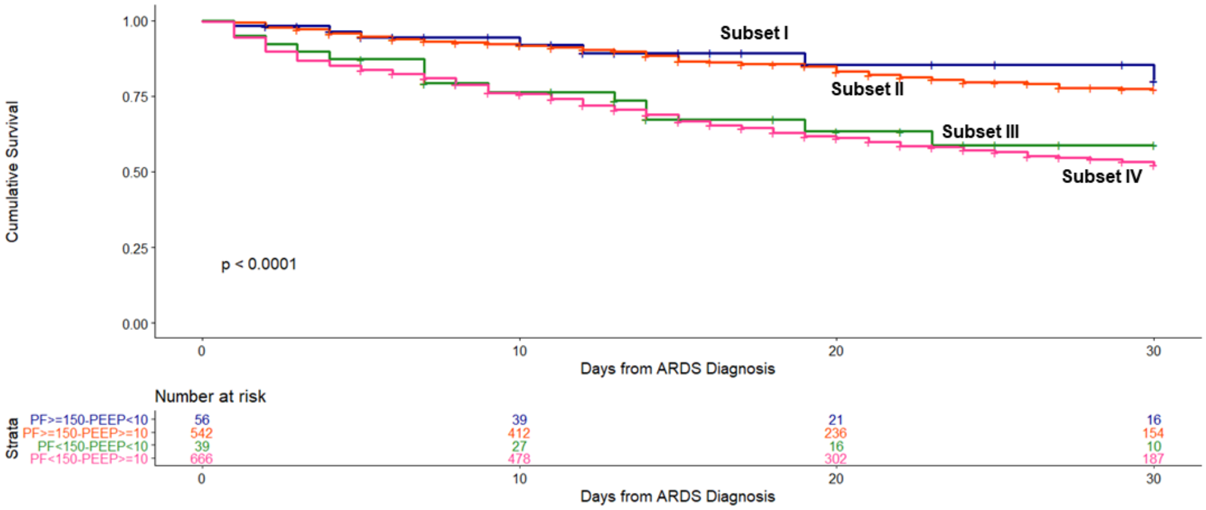

SUPPLEMENTARY DISCUSSION

We would like to emphasize that our classification system was derived from patients with moderate-to-severe ARDS while they are intubated and mechanically ventilated with a lung-protective ventilation approach. Due to the observational nature of our study design, participating centers did not report any loss of patients with moderate-to-severe ARDS during the study periods. We did not enrol patients with persistent mild ARDS (PaO_2/FiO_2 >200 mmHg during the entire ICU stay). However, we are confident that no patients with mild ARDS were excluded during our observational periods if they moved to a more severe category, although we do not have data on the precise number of those patients.

We do not believe that there is a relevant effect of calendar time on our findings. In our cohorts, we enrolled consecutive patients with the same diagnostic criteria, with essentially no exclusion criteria, within a limited range of lung severity (moderate and severe ARDS), and assessed similarly. Patients were treated in almost the same hospitals by almost the same principal investigators throughout the study years. The approach to MV was identical, thus we can expect that other changes in the way ICU patients may have been managed during the study periods would have equal effects on survival of patients in each of the four datasets. Each cohort of the pooled testing dataset and the external confirmatory cohort had a sufficient number of events (>100 ICU deaths) [23]. It is however interesting to note that the ICU mortality across the four cohorts was similar (41%, 38%, 34.4%, 37%) and not significantly different (p=0.366), as reported in the Supplementary Results section of this Supplementary File. This study was performed in a country with universal access to health care where demographics, cultural, economics, and health care system had a minimum impact during the years of the study, and where participating centers followed the same international guidelines for identification and management of patients with ARDS like in other developed countries in the world.

This type of combined analysis of several hundreds of patients from independent cohorts has been used extensively by other authors using heterogeneous populations from previous published clinical studies [29-33], some of them with a larger variation in time. Despite that in some of those publications the investigators used data from 1990 to 2008 [29], from 1998 to 2013 [30], from 1999 to 2005 [31], from 1998 to 2014 [32], or from 2007 to 2011 [33], their findings are considered relevant for current clinical practice. Periods of screening/enrolment are clearly reported in the "patient population" subsection of Supplementary Methods in this Supplementary File.

Our findings confirmed that a given standardized ventilator setting is needed to adjust for confounding by disease progression: it is seems that patients who are getting better early in the course do better, and those who decline over the first 24 hours do worse. As postulated previously by our group [4], in future therapeutic trials the goal may be to enrol severe ARDS patients within few hours after ARDS diagnosis, but our study confirmed that to guarantee that enrolled patients are representative of the target populations, randomization should not occur until patients qualify as severe ARDS under a standardized ventilator approach at 12 to 24 hours after routine intensive care. Mixing all forms of ARDS severity in a randomized clinical trial potentially introduces distortions and biases. Randomized clinical trials that simple average the effects of therapeutic interventions across all participants can muddy the results, missing positive effects in a subset of patients. If a trial does not limit the subjects to those in a higher risk category, and those with mild forms of ARDS are not evenly distributed, the trial will not verify the value of the interventions [34,35].

Our intention was not to redefine ARDS. All our patients were screened and stratified according to current ARDS criteria. However, a plethora of observational studies and clinical trials have shown that after the initial hours of routine care, many critically ill patients recover within the first 24 h time-window from their apparently “fatal condition”. In our series of 1303 patients with moderate-to-severe ARDS, only 9 patients (16%) died in the ICU from the subset I with 56 patients. It is hard to imagine that a therapeutic trial in this subset of patients will make a major impact on the outcome of ARDS!

SUPPLEMENTARY REFERENCES

1. World Medical Association. World Medical Association Declaration of Helsinki: Ethical Principles for Medical Research Involving Human Subjects. JAMA 2013; 310:2191-4.
2. von Elm E, Altman DG, Egger M, Pocock SJ, Gøtzsche PC, Vandenbroucke JP for the STROBE Initiative. The strengthening the reporting of observational studies in epidemiology (STROBE) statement: guidelines for reporting observational studies. PLoS Medicine 2007; 4:e296.
3. Villar J, Blanco J, Añón JM, Santos-Bouza A, Blanch L, Ambrós A, et al. The ALIEN study: Incidence and outcome of acute respiratory distress syndrome in the era of lung protective ventilation. Intensive Care Med 2011; 37:1932-41.
4. Villar J, Pérez-Méndez, Blanco J, Añón JM, Blanch L, Belda J, et al; Spanish Initiative for Epidemiology, Stratification, and Therapies for ARDS (SIESTA) Network. A universal definition of ARDS: the PaO_2/FiO_2 ratio under a standard ventilatory setting – a prospective, multicenter validation study. Intensive Care Med 2013; 39:583-92.
5. Villar J, Blanco J, del Campo R, Andaluz-Ojeda D. Díaz Dominguez FJ, Muriel A, et al. Assessment of PaO2/FiO2 for stratification of patients with moderate and severe acute respiratory distress syndrome. BMJ Open 2015; 5:1006812.
6. Forrester JS, Diamond GA, Swan HJC. Correlative classification of clinical and hemodynamic function after acute myocardial infarction. Am J Cardiol 1977; 39:137-45.
7. Ranieri VM, Rubenfeld GD, Thompson BT, Ferguson ND, Caldwell E, Fan E, et al. Acute respiratory distress syndrome: The Berlin definition. JAMA 2012; 307:2526-33.
8. Villar J, Ambrós A, Soler JA, Martínez D, Ferrando C, Solano R, et al. Age, PaO_2/FiO_2, and plateau pressure score: a proposal for a simple outcome score in patients with the acute respiratory distress syndrome. Crit Care Med 2016; 44:1361-9.
9. Villar J, Martín-Rodríguez C, Domínguez-Berrot AM, Fernández L, Ferrando C, Soler JA, et al. A quantile analysis of plateau and driving pressures: Effects on mortality in patients with acute respiratory distress syndrome receiving lung-protective ventilation. Crit Care Med 2017; 45:843-50.
10. Villar J, Ambrós A, Mosteiro F, Martínez D, Fernández L, Ferrando C, et al. A prognostic enrichment strategy for selection of patients with acute respiratory distress syndrome in clinical trials. Crit Care Med 2019; 47:377-85.

11. Villar J, Mora-Ordoñez JM, Soler JA, Mosteiro F, Vidal A, Ambrós A, et al. The PANDORA study: Prevalence and outcome of acute hypoxemic respiratory failure in the pre-COVID era. Crit Care Explor 2022; 4:e0684.
12. Villar J, González-Martín JM, Ambrós A, Mosteiro F, Martínez D, Fernández L, et al. Stratification for identification of prognostic categories in the acute respiratory distress syndrome (SPIRES) score. Crit Care Med 2021; 49:e920-e930.
13. Bernard GR, Artigas A, Brigham KL, Carlet J, Falke K, Hudson L, et al. The American-European Consensus Conference on ARDS. Definitions, mechanisms, relevant outcomes, and clinical trial coordination. Am J Respir Crit Care Med 1994; 149:818-24.
14. Guerin C, Thompson T, Brower R. The ten diseases that look like ARDS. Intensive Care Med 2015; 41:1099-102.
15. Acute Respiratory Distress Syndrome Network. Ventilation with lower tidal volumes as compared with traditional tidal volumes for acute lung injury and the acute respiratory distress syndrome. N Engl J Med 2000; 342:1301-8.
16. Knaus WA, Draper EA, Wagner DP, Zimmerman JE. APACHE II: a severity of disease classification system. Crit Care Med 1985; 13:818-29.
17. Villar J, Pérez-Méndez L, López J, Belda J, Blanco J, Saralegui I, et al. An early PEEP/FIO2 trial identifies different degrees of lung injury in patients with acute respiratory distress syndrome. Am J Respir Crit Care Med 2007; 176:795-804.
18. Vincent JL, de Mendonça A, Cantraine F, Moreno R, Takala J, Suter PM, el al. Use of the SOFA score to assess the incidence of organ dysfunction/failure in intensive care units: results of a multicenter, prospective study. Working group on "sepsis-related problems" of the European Society of Intensive Care Medicine. Crit Care Med 1998; 26:1793-1800.
19. Levy MM, Fink MP, Marshall JC, Abraham E, Angus D, Cook D, et al. 2001 SCCM/ESICM/ACCP/ATS/SIS International sepsis definitions conference. Crit Care Med 2003; 31:1250-56.
20. Singer M, Deutschman CS, Seymour CW, Shankar-Hari M, Annane D, Bauer M, et al. The Third International Consensus Definition for sepsis and septic shock. JAMA 2016; 315:801-10.

21. Eke G, Bloos F, Wilson DC, Meybohm P, SepNet Critical Care Trails Group. Identification of developing multiple organ failure in sepsis patients with low or moderate SOFA scores. Crit Care 2018; 22:147.
22. Yehya N, Harhay MO, Curley MAQ, Schoenfeld DA, Reeder RW. Re-appraisal of ventilator-free days in critical care research. Am J Respir Crit Care Med 2019; 200:828-36.
23. Vergouwe Y, Steyerberg EW, Eijkemans MJC, Habbema JDF. Substantial effective sample sizes were required for external validation studies of predictive logistic regression models. J Clin Epidemiol 2005; 58:475-83.
24. Eisenach JC, Kheterpal S, Houle TT. Reporting of observational research in Anesthesiology: the importance of the analysis plan. Anesthesiology 2016; 124:998-1000.
25. Ioannidis JPA. The proposal to lower P value thresholds to 0.005. JAMA 2018; 319:1429-30.
26. Tonelli AR, Zein J, Adams J, Ioannidis JPA. Effects of interventions on survival in acute respiratory distress syndrome: an umbrella review of 159 published randomized trials and 29 meta-analyses. Intensive Care Med 2014; 40:769-87.
27. Detsky AS. Learning the art and science of diagnosis. JAMA 2022; 327:1759-60.
28. Bellani G, Laffey JG, Pham T, Fan E, Brochard L, Esteban A, et al. Epidemiology, patterns of care, and mortality for patients with acute respiratory distress syndrome in intensive care units in 50 countries. JAMA 2016; 315:788-800.
29. Amato MB, Meade MO, Slutsky AS, Brochard L, Costa EL, Schoenfeld DA, et al. Driving pressure and survival in the acute respiratory distress syndrome. N Engl J Med 2015; 372:747-55.
30. Rautanen A, Mills TC, Gordon AC, Hutton P, Steffens M, Nuamah R, et al. Genome-wide association study of survival from sepsis due to pneumonia: and observational cohort study. Lancet Respiratory Med 2015; 3:53-60.
31. Metkus TS, Guallar E, Sokoll L, Morrow D, Tomaselli G, Brower R, et al. Prevalence and prognostic association of circulating troponin in acute respiratory distress syndrome. Critical Care Med 2017; 45:1709-17.
32. Zhang R, Wang Z, Tejera P, Frank AJ, Wei Y, Su L, et al. Late-onset moderate to severe acute respiratory distress syndrome is associated with shorter survival and higher mortality: a two-stage association study. Intensive Care Med 2017; 43:399-407.

33. Brown SM, Wilson EL, Presson AP, Dinglas VD, Greene T, Hopkins RO, et al. Understanding patient outcomes after acute respiratory distress syndrome: identifying subtypes of physical cognitive and mental health outcomes. Thorax 2017; 72:1094-103.
34. Villar J, Pérez-Méndez L, Aguirre-Jaime A, Kacmarek RM. Why are physicians so skeptical about positive randomized controlled clinical trials in critical care medicine. Intensive Care Med 2005; 31:196-204.
35. Villar J, Ferrando C, Tusman G, Berra L, Rodríguez-Suárez P, Suárez-Sipmann F. Unsuccessful and successful clinical trials in acute respiratory distress syndrome: addressing physiology-based gaps. Front Physiol 2021; 12:774025.
